# Genomic surveillance of Rift Valley fever virus: from sequencing to lineage assignment

**DOI:** 10.1186/s12864-022-08764-6

**Published:** 2022-07-18

**Authors:** John Juma, Vagner Fonseca, Samson L. Konongoi, Peter van Heusden, Kristina Roesel, Rosemary Sang, Bernard Bett, Alan Christoffels, Tulio de Oliveira, Samuel O. Oyola

**Affiliations:** 1 International Livestock Research Institute (ILRI), Nairobi, Kenya; 2 South African MRC Bioinformatics Unit, South African National Bioinformatics Institute, Cape Town, South Africa; 3 KwaZulu-Natal Research Innovation and Sequencing Platform (KRISP), School of Laboratory Medicine and Medical Sciences, College of Health Sciences, University of KwaZulu-Natal, Durban, South Africa; 4 Centre for Epidemic Response and Innovation (CERI), School of Data Science and Computational Thinking, Stellenbosch University Stellenbosch, Stellenbosch, South Africa; 5 Laboratorio de Genética Celular e Molecular, Instituto de Ciências Biologicas, Universidade Federal de Minas Gerais, Belo Horizonte, Minas Gerais Brazil; 6 Organização Pan-Americana da Saúde/Organização Mundial da Saúde, Brasília, Distrito Federal Brazil; 7 Kenya Medical Research Institute (KEMRI), Nairobi, Kenya; 8 Centre for the AIDS Programme of Research in South Africa (CAPRISA), Durban, South Africa; 9 Department of Global Health, University of Washington, Seattle, WA USA

**Keywords:** RVFV, Rift Valley fever virus, Genotyping, Genomic surveillance, Lineage, L-segment, M-segment, S-segment, Glycoprotein Gn, Sequencing

## Abstract

Genetic evolution of Rift Valley fever virus (RVFV) in Africa has been shaped mainly by environmental changes such as abnormal rainfall patterns and climate change that has occurred over the last few decades. These gradual environmental changes are believed to have effected gene migration from macro (geographical) to micro (reassortment) levels. Presently, 15 lineages of RVFV have been identified to be circulating within the Sub-Saharan Africa. International trade in livestock and movement of mosquitoes are thought to be responsible for the outbreaks occurring outside endemic or enzootic regions. Virus spillover events contribute to outbreaks as was demonstrated by the largest epidemic of 1977 in Egypt. Genomic surveillance of the virus evolution is crucial in developing intervention strategies. Therefore, we have developed a computational tool for rapidly classifying and assigning lineages of the RVFV isolates. The computational method is presented both as a command line tool and a web application hosted at https://www.genomedetective.com/app/typingtool/rvfv/. Validation of the tool has been performed on a large dataset using glycoprotein gene (Gn) and whole genome sequences of the Large (L), Medium (M) and Small (S) segments of the RVFV retrieved from the National Center for Biotechnology Information (NCBI) GenBank database. Using the Gn nucleotide sequences, the RVFV typing tool was able to correctly classify all 234 RVFV sequences at species level with 100% specificity, sensitivity and accuracy. All the sequences in lineages A (*n* = 10), B (*n* = 1), C (*n* = 88), D (*n* = 1), E (*n* = 3), F (*n* = 2), G (*n* = 2), H (*n* = 105), I (*n* = 2), J (*n* = 1), K (*n* = 4), L (n = 8), M (*n* = 1), N (*n* = 5) and O (*n* = 1) were also correctly classified at phylogenetic level. Lineage assignment using whole RVFV genome sequences (L, M and S-segments) did not achieve 100% specificity, sensitivity and accuracy for all the sequences analyzed. We further tested our tool using genomic data that we generated by sequencing 5 samples collected following a recent RVF outbreak in Kenya. All the 5 samples were assigned lineage C by both the partial (Gn) and whole genome sequence classifiers. The tool is useful in tracing the origin of outbreaks and supporting surveillance efforts.

Availability: https://github.com/ajodeh-juma/rvfvtyping

**Supplementary Information:**

The online version contains supplementary material available at 10.1186/s12864-022-08764-6.

## Introduction

Rift Valley fever (RVF) is an acute febrile mosquito-borne zoonotic disease caused by the Rift Valley fever virus (RVFV) [1]. The disease primarily affects animals and humans and is responsible for deaths in human and livestock populations. It leads to major losses in livestock production, thus negatively affecting livelihoods in Sub-Saharan Africa [2]. It is a well-known livestock disease in Africa and Arabian Peninsula that is linked with epizootic and epidemic events [3]. In animals, it is usually characterized by high mortality and abortion rates in a phenomenon termed ‘abortion storm’ [1]. In humans, RVF presents itself with clinical signs ranging from mild to severe [2, 4, 5]. Severe symptoms vary although typical signs include retinitis, hepatitis, delayed onset encephalitis and hemorrhagic disease. The overall case fatality ratio is estimated to be between 0.5 and 2.0% [2]. RVF was first characterized in 1931 in the Great Rift Valley region of Kenya following an epidemic among sheep [6].

Circulation of RVFV in majority of African countries and a few in the Middle East has been reported through serological surveys, animal and human cases as well as outbreak reports [7–9]. As a result of the increasing spread of the virus outside its endemic settings, high number of competent vectors, increased international trade in livestock and climate change, there is need for coordinated efforts to better prepare for a possible emergence of RVF in disease-free countries [2]. RVF has been identified and listed by the World Health Organization (WHO) as likely to cause future epidemics in a new emergency plan developed after the Ebola epidemics of 2018 [10].

Overall, RVFV genome has been shown to be highly conserved as elucidated by sequencing and phylogenetic studies [11–13]. Irrespective of the genomic segment, the diversity at nucleotide and amino acid levels have been reported to be approximately 4 and 1% respectively [2, 14]. Variations within the genome occur as random single nucleotide polymorphisms (SNPs) with no well-defined variable sites. This makes it difficult to differentiate between strains without genome sequencing since there are no well-defined serotypes [15].

Genomic surveillance has become a critical tool for elucidating genetic diversity of viruses and is crucial in understanding the emergence and spread of outbreaks. This is particularly important for the development of effective intervention and prevention measures including diagnosis and vaccine initiatives. Moreover, when such a surveillance is undertaken at fine resolution with consistent classification of reported sequences, strains linked with greater epidemic potential or disease severity can be detected and characterized. There is need for methods that can reliably classify arboviruses based on their genome sequences. In addition, whole genome sequences are often lacking in routine clinical settings. In turn, short gene sequences are often used to attain classification and lineage assignment at viral species level [16]. Here we present a computational method for lineage assignment of RVFV sequences. The lineage assignment method is implemented both as a web application and command line tool. The web-based method is built on top of a Genome Detective software tool [17] while the command line is implemented in Nextflow language [18], that is both scalable and reproducible. The method was validated with a dataset comprising of 234 samples using both partial and whole genome sequences. The tool was further evaluated using genomic sequences generated from a recent RVF outbreak in Kenya.

## Methods

### Lineage assignment and classification

**Fig. 1:**
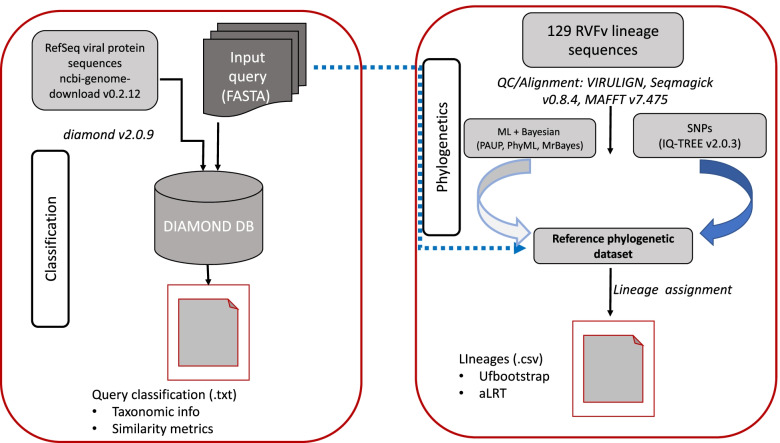
Schematic representation of the command line workflow. The workflow begins with virus classification using DIAMOND and reports the output as a text file with taxonomic information and similarity metrics. Phylogenetic analysis is performed using a default phylogenetic reference dataset generated by Neighbor-Joining (NJ), Maximum likelihood (ML) and Bayesian tree. Users can specify which phylogenetic reference dataset to use. Query sequences are aligned to the reference dataset multiple sequence alignment with MAFFT, and a ML phylogenetic tree is constructed followed by lineage assignment. An output file with the lineage assignment, bootstrap values and likelihood test ratio is generated in comma-separated values (CSV) file format

We developed a method that allows for classification and lineage assignment of consensus partial sequences (glycoprotein gene, Gn) and whole genome sequences (complete L, M and S-segments) (Fig. 1). To build a database for virus species assignment, we downloaded 10,368 (as of 29th May 2021) virus genome sequences from NCBI RefSeq database [19]. This translated to 501,622 protein sequences. A local database was constructed using DIAMOND [20] with the provision of taxon names, nodes and protein accession to taxonomic identifier files obtained from NCBI.

#### Glycoprotein, Gn classifier dataset

Applying the nomenclature implemented in the largest phylogenetic study conducted by Grobbelaar and colleagues [13], we identified representative taxa for each lineage that we used to build the RVFV Typing tool. Sequences were compiled from the NCBI nucleotide database [21]. This dataset - used as reference - comprised of 129 partial and whole genome sequences for the M-segment. The annotation of original location, collection date as well as the originating and submitting laboratory or data generators are shown in Table S5. These sequences were deduplicated on sequence composition and an alignment was constructed with MAFFT [22]. Each alignment was edited manually until a codon-correct consensus sequence between positions 815 and 1306 was achieved using seqmagick with the option --mask 1:815,1306:3885. These coordinates represent the start and the end positions of the 490 bp M-segment glycoprotein gene (Gn). The suitability of the M-segment was chosen due to its relatively high variability 2 and 5% at the amino acid and nucleotide levels respectively compared to other segments [15]. The M-segment also encodes for surface glycoproteins which are targets for neutralizing antibodies and play essential role in virus attachment. Given this role, positive selective pressure is expected to be responsible for the evolutionary patterns observed in the Gn gene [23].

#### Lineage delineation using single nucleotide polymorphisms (SNPs)

In order to generate representative dataset to be used for lineage assignment using phylogenetic inference, we delineated the lineages using SNPs. For each lineage sequence, we identified defining SNPs (i.e., those that are shared within a lineage) using M-segment reference sequence (NC_014396). Defining SNPs per lineage were considered if they were present in 90% of all the available sequences per lineage. A target of 5 sequences or more per lineage was aimed at although some lineages contained only single isolate sequences. Lineages with less than 5 sequences were all included into the representative dataset. This resulted into 51 unique representative sequences referred to as the Gn classifier. The next step in this exploration involved a phylogenetic analysis. We identified a suitable substitution model with consistent tree topologies using modeltest-ng [24]. Using a general time reversible (GTR) [25] with discrete gamma distributed rate variation among sites as the optimal model which gave consistent tree topologies with IQ-TREE [26], we constructed a phylogenetic tree using IQ-TREE [26] (i.e., Maximum likelihood, 1000 ultrafast bootstrap approximation with 1000 likelihood ratio test) to generate a consensus phylogenetic tree.

#### Complete segments classifier datasets

In order to generate lineage assignment representative SNPs for the whole genome segments, we retrieved 408 sequences from NCBI. Out of the 408, only 234 had complete sequences available for each of the 3 segments. For L, M and S-segments, we used NC_014397, NC_014396 and NC_014395 as reference sequences respectively. We used the 234 sequences to build representative lineage assignment SNPs from which we identified 47 unique representative sequences for lineage assignment. The 47 sequences were aligned using MAFFT [22] and manually edited followed by construction of a Maximum likelihood phylogenetic tree using a generalized time reversible substitution model with invariable sites and discrete gamma distribution (GTR + I + G4) [25].

### Sample processing and genomic sequencing of RVF outbreak isolates

For the new samples collected from a recent outbreak in Kenya, detailed protocols used for sample collection and processing, ELISA analysis, virus isolation through culture, sequencing library preparation and genome assembly are provided as supplementary material.

#### The web application

Representative sequences used in the web interface were identified using bootstrap support and posterior probability values. These values were obtained from a maximum likelihood phylogenetic tree using PhyML [27] and a Bayesian tree constructed using MrBayes [28]. The trees were visualized in Figtree [29]. We selected 53 reference sequences that represent the diversity of each of the lineages. Taxa with bootstrap support of 100% and posterior probability of 1 were used as the criteria in the selection of reference sequences. The phylogenetic reference dataset using the representative sequences was used to create an automated RVFV Typing Tool. We selected 5–10 sequences that represented the diversity of each virus lineage. We included all the sequences in a lineage where the total number of available sequences was less than 5. Sequences that met these selection criteria were quality checked for the presence of insertions, deletions, frame shifts and non-IUPAC characters using VIRULIGN [30]. Sequences that passed the quality control were aligned using MAFFT [22], and were subjected to phylogenetic analysis using PAUP* (i.e. Neighbour Joining), MrBayes (i.e. Bayesian) and PhyML (i.e. Maximum likelihood) [27, 28, 31, 32] using GTR + G + I [25]. Sequences that gave consistent topologies using all three tree inference methods were retained as potential reference sequences and used in the next step of the evaluation process.

#### Similarity search and lineage assignment

In both implementations, sequence classification and lineage assignment involve a similarity search against a viral protein database using the RVFV whole/partial genome nucleotide sequences as query followed by phylogenetic analysis. Classification of query sequences was performed using DIAMOND BLASTX. DIAMOND is a high-throughput program for aligning sequences with high sensitivity against a protein reference database and is up to 20,000 times the speed of BLAST. Phylogenetic analysis for lineage assignment was achieved by construction of Maximum likelihood phylogenetic tree using IQ-TREE. This process was achieved by obtaining an alignment of the query against the reference dataset using the option --add in MAFFT. A Maximum likelihood phylogenetic tree was constructed using the GTR + G4 distance metric with 1000 bootstrap replicates. Single branch tests using the SH-like approximate likelihood ratio test was performed to assess the bootstrap support values. Polytomies were collapsed if the branch lengths were below a given threshold (default 0.000005). The query sequence was assigned to a particular lineage if it clusters monophyletically with that genotype clade with ultrafast bootstrap support > 70%. Query sequences whose values were below this cut-off were reported as unassigned.

The web implementation of lineage assignment involved construction of a Neighbour Joining (NJ) phylogenetic tree that was used to make assignments at the lineages level. For this component, the query sequence was aligned against a set of reference sequences using the profile alignment option in the ClustalW software [33], such that the query sequence was added to the existing alignment of reference sequences. Following the alignment, a NJ phylogenetic tree, with 100 bootstrap replicates was inferred. The tree was constructed using the HKY [34] distance metric with gamma among-site rate variation, as implemented in the PAUP* software [31]. The query sequence was assigned to a particular lineage if it clusters monophyletically with a genotype clade with bootstrap support > 70%. If the bootstrap support was < 70%, the genotype was reported to be unassigned. This web interface is built using the Genome detective framework [17].

**Fig. 2:**
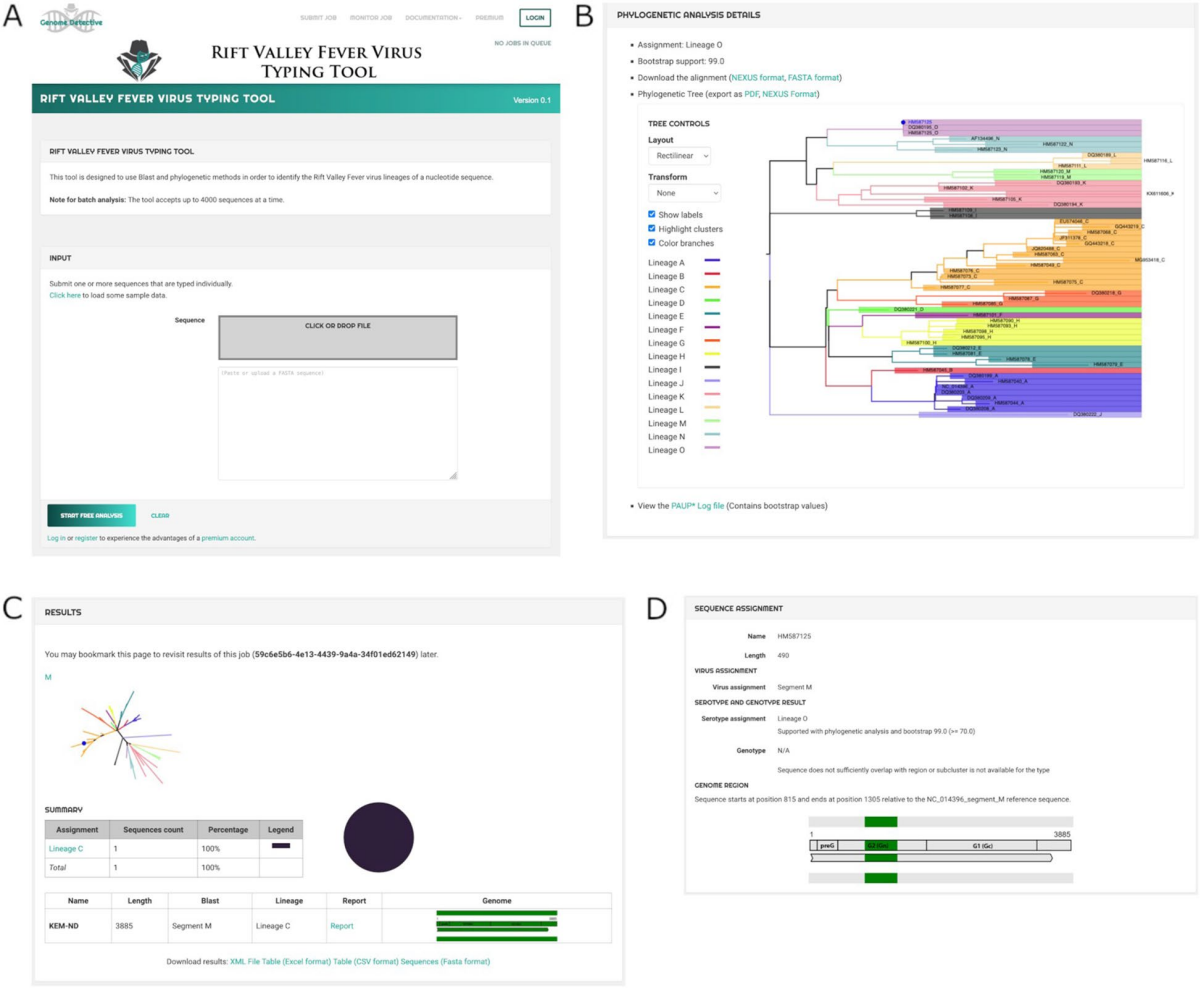
Screenshot of the web interface for RVFV typing tool. (**A**) The web interface offers a portal for users to perform classification and visualize the results. The typing report provides information on the sequence name of the query sequence, the nucleotide length of the sequence, an illustration of the position in the virus’ genomic segment, the species assignment and the genotype assignment. A detailed report (**B**) is provided for the phylogenetic analysis that resulted into this classification. All results can be exported to a variety of file formats (XML, CSV, Excel or FASTA format). The detailed HTML report (**C**) contains information on the sequence name, length, assigned virus and genotype, an illustration (**D**) of the position of the sequence in the virus’ genomic segment and the phylogenetic analysis section. The alignment section shows the alignment and constructed phylogenetic tree

Both the command line and the web application tool produce classification and phylogenetic lineage assignment results as report text files. The report includes static (for the command line) and interactive (for the web application) phylogenetic trees as data visualization output (Fig. 2).

#### Performance evaluation matrix

The True positive rate (TPR) or sensitivity, False positive rate (FPR) or specificity and accuracy of our proposed method were computed for both the assignment of species and phylogenetic clustering. TPR/Sensitivity was computed by the formula $\frac{TP}{TP+ FN}$, FPR/specificity by $\frac{FP}{FP+ TN}$ and accuracy by $\frac{TP+ TN}{TP+ TN+ FP+ FN}$, where TP = True Positives, FP = False Positives, TN = True Negatives and FN = False Negatives.

## Results

### Identification of lineage assignment SNPs

In searching for RVFV sequences on the NCBI database, we observed a general paucity in availability of the virus sequence data. The number of viral sequences per country also varied, with South Africa, Kenya, Zimbabwe and Egypt having the majority (> 10). The variation in the number of sequences available in public databases can be attributed to the frequency of outbreaks and the effort by individual countries and their partners to sequence and report the isolates (Fig. 4). For the purposes of identifying lineage assignment SNPs, we 129 RVF virus sequences. For each of the four lineage classifying sequence datasets (Gn, S, M and L sequences), we identified lineage defining SNPs for all the available sequences. For the Gn sequences, we identified a total of 121 lineage assignment SNPS distributed across all the 15 RVFV lineages. Table 1 shows the Gn lineage assignment SNPs while those identified in S, M and L whole genome segment sequences are listed in Tables S1, S2 and S3 respectively.

**Table 1:** RVFV Lineage defining single nucleotide polymorphisms (SNPs) in Glycoprotein (Gn) gene. For each lineage sequences, SNPs were identified in comparison to the reference (strain ZH-548). Since the reference strain falls within lineage A, there were no observed SNPs in the category

Lineage	SNPs	Total
A		1
B	830GA;1103TC;1142TC;1304GA	4
C	836TA;926GA;1103TC;1163CT;1190TC;1241AG	6
D	839TC;926GA;1103TC;1142TC;1163CT;1195GA	6
E	854TA;926GA;1103TC;1142TC;1163CT;1166AG	6
F	816AG;902GA;926GA;1079GA;1103TC;1106GA;1142TC;1163CT;1253GA	9
G	926GA;1103TC;1142TC;1163CT	4
H	920AG;926GA;1103TC;1142TC;1157AG;1163CT;1169AT	7
I	833CT;920AG;986CT;998TC;1049GA;1103TC;1115GA;1142TC;1163CT;1304GA	10
J	836TC;860CT;920AG;926GA;953AG;995GA;1007CA;1055TC;1115GA;1142TC;1154GA;1160GA;1161TC;1163CT;1190TC;1250TC	16
K	894CT;1091TC;1115GA;1142TC;1250TC	5
L	842GA;866CT;917CT;920AG;926GA;1103TC;1115GA;1122CT;1124AG;1142TC;1163CT;1190TC;1250TC;1274AT;1304GA	15
M	857GA;894CT;920AG;924TC;926GA;992GT;1103TC;1115GA;1142TC;1151TC;1163CT;1250TC;1304GA	13
N	920AG;926GA;1103TC;1112GA;1115GA;1142TC;1163CT;1187GA;1304GA	9
O	920AG;926GA;1103TC;1106GA;1115GA;1142TC;1163CT;1205AG;1243AG;1250TC;1304GA	11

### Maximum likelihood phylogenetic trees for lineage clustering

**Fig. 3:**
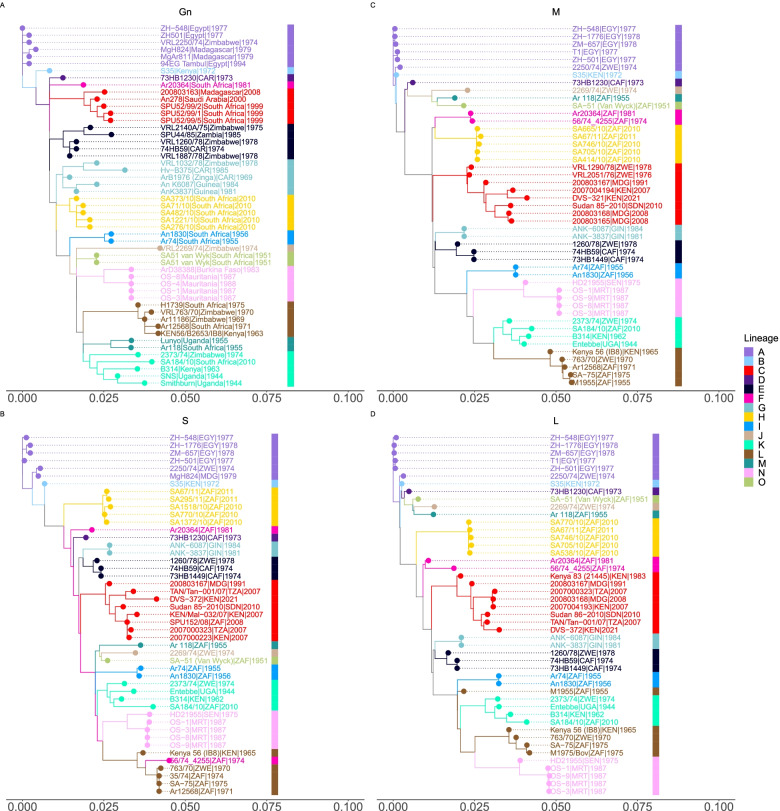
Phylogenetic analysis using Gn and whole genome (L, M & S) segment classifiers. **A**-**D** Maximum likelihood (ML) phylogenetic trees inferred from the representative sequences for all lineages within the (A) 51 sequences of the glycoprotein (490 bp) gene aligned with MAFFT and ML tree inferred under the GTR + I + G substitution model, (B) 47 sequences of the Small (S) segment (1690 bp), (C) 47 sequences of the Medium (M) segment (3885 bp) and (D) 47 sequences of the Large (L) segment (6404 bp). All the trees show similar topology for all the lineages

Using the unique representative sequences for both Gn (*n* = 51) and whole genome sequences (*n* = 47) for the three segments (L, M & S), we constructed maximum likelihood phylogenetic trees shown in Fig. 3. We observed stricking similarity in the tree topology generated with both the glycoprotein (Gn) gene and with the RVFV whole genome sequences used. As is expected, each lineage formed a distinct cluster shown as monophyletic clades (Fig. 3). This indicates successful classification by the assignment tool.

### Evaluating of lineage assignment using the glycoprotein gene (Gn) as classifier

In order to determine the accuracy of the tool and applying the nomenclature implemented by Grobbelaar [13], we used a total of 129 partial sequences spanning the Gn gene out of which 51 were the unique representative dataset used as lineage defining sequences in developing the tool.

All the 129 RVFV sequences distributed in lineages A (*n* = 13), B (*n* = 1), C (*n* = 44), D (*n* = 1), E (*n* = 7), F (*n* = 1), I (*n* = 2), J (*n* = 1), M (*n* = 2), N (*n* = 13) and O (*n* = 2) were correctly classified at phylogenetic level, with accuracy, sensitivity and specificity of 100%. We also obtained near perfect classification (i.e., 99%) at phylogenetic level for lineages G (*n* = 8) and H (*n* = 13). Only one sequence (accession HM587100) could not be assigned as per its original lineage assignment. Upon exclusion of HM587100 sequence, the typing tool assigned all the remaining 128 sequences with 100% sensitivity and accuracy. Representative sequences belonging to lineage G (HM587087, HM587083, AF134499, DQ380218) and J (DQ380222) were correctly assigned but with low bootstrap support values below the set threshold of 70%. A detailed classification performance is shown in Table 2.

**Table 2:** Validation/testing of the RVFV Typing tool to classify partial and whole genome sequences (*n* = 128) using glycoprotein sequences. The classification results were compared to manual phylogenetic analysis. Abbreviations as used in this table: *TP* True Positives, *TN* True Negatives, *FP* False Positives, *FN* False Negatives, *TPR* True Positive Rate, *FPR* False Positive Rate, *ACC* Accuracy

Lineage	Known	TP	TN	FP	FN	TPR	FPR	ACC
A	13	13	115	0	0	100.0	0.0	100.0
B	1	1	127	0	0	100.0	0.0	100.0
C	44	44	84	0	0	100.0	0.0	100.0
D	1	1	127	0	0	100.0	0.0	100.0
E	7	7	121	0	0	100.0	0.0	100.0
F	1	1	127	0	0	100.0	0.0	100.0
G	8	8	120	0	0	100.0	0.0	100.0
H	12	12	116	0	0	100.0	0.0	100.0
I	2	2	126	0	0	100.0	0.0	100.0
J	1	1	127	0	0	100.0	0.0	100.0
K	11	11	117	0	0	100.0	0.0	100.0
L	10	10	118	0	0	100.0	0.0	100.0
M	2	2	126	0	0	100.0	0.0	100.0
N	13	13	115	0	0	100.0	0.0	100.0
O	2	2	126	0	0	100.0	0.0	100.0

### Evaluating lineage assignment using whole RVFV genome sequences (L, M and S-segments) as classifiers

We further assessed the performance of the tool using lineage classification nomenclature proposed by Grobbelaar [13]. For this assessment we used 234 whole genome sequences. Most of the sequences were correctly assigned at 100% accuracy. However, a few sequences, most of which had only a single sequence per lineage were assigned at 99% accuracy as shown in Tables 3, 4 and 5 for L, M and S-segments respectively. However, using the glycoprotein gene (Gn) sequence obtained from the 234 whole genome sequences, we performed lineage assignment and obtained 100% accuracy for all the sequences (Table 6).

**Table 3:** Validation/testing of the RVFV Typing tool to classify whole genome sequences (*n* = 234) using complete L-segment sequences. The classification results were compared to manual phylogenetic analysis. Abbreviations as used in this table: *TP* True Positives, *TN* True Negatives, *FP* False Positives, *FN* False Negatives, *TPR* True Positive Rate, *FPR* False Positive Rate, *ACC* Accuracy

Lineage	Known	TP	TN	FP	FN	TPR	FPR	ACC
A	10	11.0	223.0	1.0	0.0	100.0	0.45	99.57
B	1	1.0	233.0	0.0	0.0	100.0	0.0	100.0
C	88	93.0	141.0	5.0	0.0	100.0	3.42	97.91
D	1	0.0	234.0	0.0	1.0	0.0	0.0	99.57
E	3	3.0	231.0	0.0	0.0	100.0	0.0	100.0
F	2	1.0	233.0	0.0	1.0	50.0	0.0	99.57
G	2	2.0	232.0	0.0	0.0	100.0	0.0	100.0
H	105	99.0	135.0	0.0	6.0	94.29	0.0	97.5
I	2	3.0	231.0	1.0	0.0	100.0	0.43	99.57
J	1	0.0	234.0	0.0	1.0	0.0	0.0	99.57
K	4	6.0	228.0	2.0	0.0	100.0	0.87	99.15
L	8	10.0	224.0	2.0	0.0	100.0	0.88	99.15
M	1	0.0	234.0	0.0	1.0	0.0	0.0	99.57
N	5	5.0	229.0	0.0	0.0	100.0	0.0	100.0
O	1	0.0	234.0	0.0	1.0	0.0	0.0	99.57

**Table 4:** Validation/testing of the RVFV Typing tool to classify whole genome sequences (*n* = 234) using complete M-segment representative sequences. The classification results were compared to manual phylogenetic analysis. Abbreviations as used in this table: *TP* True Positives, *TN* True Negatives, *FP* False Positives, *FN* False Negatives, *TPR* True Positive Rate, *FPR* False Positive Rate, *ACC* Accuracy

Lineage	Known	TP	TN	FP	FN	TPR	FPR	ACC
A	10	12.0	222.0	2.0	0.0	100.0	0.89	99.15
B	1	0.0	234.0	0.0	1.0	0.0	0.0	99.57
C	88	89.0	145.0	1.0	0.0	100.0	0.68	99.57
D	1	0.0	234.0	0.0	1.0	0.0	0.0	99.57
E	3	3.0	231.0	0.0	0.0	100.0	0.0	100.0
F	2	4.0	230.0	2.0	0.0	100.0	0.86	99.15
G	2	2.0	232.0	0.0	0.0	100.0	0.0	100.0
H	105	102.0	132.0	0.0	3.0	97.14	0.0	98.73
I	2	4.0	230.0	2.0	0.0	100.0	0.86	99.15
J	1	0.0	234.0	0.0	1.0	0.0	0.0	99.57
K	4	5.0	229.0	1.0	0.0	100.0	0.43	99.57
L	8	8.0	226.0	0.0	0.0	100.0	0.0	100.0
M	1	0.0	234.0	0.0	1.0	0.0	0.0	99.57
N	5	5.0	229.0	0.0	0.0	100.0	0.0	100.0
O	1	0.0	234.0	0.0	1.0	0.0	0.0	99.57

**Table 5:** Validation/testing of the RVFV Typing tool to classify whole genome sequences (*n* = 234) using complete S-segment sequences. The classification results were compared to manual phylogenetic analysis. Abbreviations as used in this table: *TP* True Positives, *TN* True Negatives, *FP* False Positives, *FN* False Negatives, *TPR* True Positive Rate, *FPR* False Positive Rate, *ACC* Accuracy

Lineage	Known	TP	TN	FP	FN	TPR	FPR	ACC
A	10	11.0	223.0	1.0	0.0	100.0	0.45	99.57
B	1	1.0	233.0	0.0	0.0	100.0	0.0	100.0
C	88	88.0	146.0	0.0	0.0	100.0	0.0	100.0
D	1	1.0	233.0	0.0	0.0	100.0	0.0	100.0
E	3	5.0	229.0	2.0	0.0	100.0	0.87	99.15
F	2	2.0	232.0	0.0	0.0	100.0	0.0	100.0
G	2	0.0	234.0	0.0	2.0	0.0	0.0	99.15
H	105	103.0	131.0	0.0	2.0	98.1	0.0	99.15
I	2	2.0	232.0	0.0	0.0	100.0	0.0	100.0
J	1	1.0	233.0	0.0	0.0	100.0	0.0	100.0
K	4	5.0	229.0	1.0	0.0	100.0	0.43	99.57
L	8	7.0	227.0	0.0	1.0	87.5	0.0	99.57
M	1	1.0	233.0	0.0	0.0	100.0	0.0	100.0
N	5	5.0	229.0	0.0	0.0	100.0	0.0	100.0
O	1	2.0	232.0	1.0	0.0	100.0	0.43	99.57

**Table 6:** Validation/testing of the RVFV Typing tool to classify whole genome sequences (*n* = 234) using partial glycoprotein representative sequences. The classification results were compared to manual phylogenetic analysis. Abbreviations as used in this table: *TP* True Positives, *TN* True Negatives, *FP* False Positives, *FN* False Negatives, *TPR* True Positive Rate, *FPR* False Positive Rate, *ACC* Accuracy

Lineage	Known	TP	TN	FP	FN	TPR	FPR	ACC
A	10	10	224	0	0	100.0	0.0	100.0
B	1	1	233	0	0	100.0	0.0	100.0
C	88	88	146	0	0	100.0	0.0	100.0
D	1	1	233	0	0	100.0	0.0	100.0
E	3	3	231	0	0	100.0	0.0	100.0
F	2	2	232	0	0	100.0	0.0	100.0
G	2	2	232	0	0	100.0	0.0	100.0
H	105	105	129	0	0	100.0	0.0	100.0
I	2	2	232	0	0	100.0	0.0	100.0
J	1	1	233	0	0	100.0	0.0	100.0
K	4	4	230	0	0	100.0	0.0	100.0
L	8	8	226	0	0	100.0	0.0	100.0
M	1	1	233	0	0	100.0	0.0	100.0
N	5	5	229	0	0	100.0	0.0	100.0
O	1	1	233	0	0	100.0	0.0	100.0

### Lineage assignment of a recent RVF outbreak in Kenya

We further used the assignment tool to analyze RVFV sequences generated from clinical livestock samples that were collected from a recent RVF outbreak in Kenya. Using IgM capture ELISA method, 5 samples were positive indicating a recent infection with RVFV (Table S4). These samples also showed low cycle threshold (Ct) values (ranging from 14 to 19) on RT-qPCR indicating sufficient viral load for whole genome sequencing (Fig. S2). The 5 samples produced whole genome sequences with a coverage of over 99% (Fig. S2). The sequences were subjected to lineage assignment and classification using the glycoprotein gene and whole genome sequences (S, M and L complete segments) classifiers. Both the glycoprotein gene and whole genome classifiers assigned all the 5 sequences to lineage C (Table 7).

**Table 7:** RVFV Typing tool lineage assignment analysis. Tabular results of the phylogenetic lineage assignment analysis of query sequences. The following terminologies are used: Query, sequence identifier/header in the FASTA file; Lineage, assigned/identified lineage of the query sequence; Bootstrap, ultrafast bootstrap approximation support value; Length, length of the nucleotide sequence; Year_first; Year when the lineage was first reported; Year_last: Year when the lineage was last reported, Countries: Countries where the identified lineage have also been reported

Query	Lineage	Bootstrap	Length	Year_first	Year_last	Countries
DVS-372	C	98	3885	1976	2016	South Africa; Mauritania; Zimbabwe; Uganda; Somalia; Angola; Madagascar; Sudan; Saudi Arabia; Kenya
DVS-333	C	97	3885	1976	2016	South Africa; Mauritania; Zimbabwe; Uganda; Somalia; Angola; Madagascar; Sudan; Saudi Arabia; Kenya
DVS-356	C	91	3885	1976	2016	South Africa; Mauritania; Zimbabwe; Uganda; Somalia; Angola; Madagascar; Sudan; Saudi Arabia; Kenya
DVS-321	C	93	3885	1976	2016	South Africa; Mauritania; Zimbabwe; Uganda; Somalia; Angola; Madagascar; Sudan; Saudi Arabia; Kenya
DVS-230	C	96	3885	1976	2016	South Africa; Mauritania; Zimbabwe; Uganda; Somalia; Angola; Madagascar; Sudan; Saudi Arabia; Kenya

## Discussion

RVFV has been shown to have low genomic diversity at both nucleotide and amino acid levels [11, 13]. However, the M-segment of the virus has been reported to be slightly more diverse at 5 and 2% in the nucleotide and amino acid levels respectively, compared to the L and S-segments shown to have compositional differences at the nucleotide and amino acid levels of 4 and 1% respectively [11, 13]. The observed limited diversity in the virus suggests that it may have a low tolerance for mutation [11]. This limited diversity has been captured by our proposed RVFV lineage assignment tool which delineated the clades based on SNPs. The observation of common mutations across multiple lineages also pinpoints the low mutation rate within the RVFV genome. Delineating lineages using SNPs highlighted the impact of shared or common mutations in the lineage assignment process. The presence of common SNPs reduces the sensitivity of lineage assignment. For instance, lineage defining representative sequences in the complete L-segment showed that there were 39 SNPs common to lineages H and C, 126 SNPs common to lineages J and I and 115 SNPs common to lineages O and L. Representative sequences in the complete M-segment showed that 33 SNPs are common to lineages D and C, 13 SNPs common to lineage H and F, 77 SNPs common to lineage J and I, 87 SNPs common to lineage M and K, and 83 SNPs common to lineage O and I. For the complete S-segment representative sequences, 31 SNPs are common to lineages G and E, and 33 SNPs common to lineage O and L. Shared mutations between two or more lineages makes it difficult to identify definitive mutations that can be confidently used in lineage assignment. This was a common occurrence in using whole genome sequence analysis to distinguish lineages H and C in L and M-segment and lineages G and E in the S-segment.

For the glycoprotein gene (Gn) classifier, the impact of common mutations was also notable in lineage assignment as illustrated in lineages G and H. The presence of shared SNPs in these lineages at positions 926 (G- > A), 1103 (T- > C), 1142 (T- > C) and 1163 (C- > T), reduced the sensitivity of the classification due to low support values.

Based on the lineage classification proposed by Grobbelaar [13], lineage assignment using RVFV whole genome sequences for L, M & S-segments was relatively less optimal with specificity (FPR – False Positive Rate) values ranging between 0.4–3.4%, sensitivity (TPR – True Positive Rate) ranging between 50 and 98% and accuracy ranging from 99 to 100% as shown in Tables 3, 4, & 5 respectively. The less optimal assignment of lineages observed with using whole genome sequences can be, to an extent, attributed to the presence of common SNPs among different lineages. Bird et al. [11] analysis produced only 7 lineages using whole genome sequences of all the segments, however Grobbelaar et al. later generated 15 lineages using the partial Gn sequences and included a South African isolate of 2010 classified as lineage H. In evaluating our tool, we have mainly compared its performance with the latest classification by Grobbelaar et al. [13] which produced the highest number of lineages.

Although whole genome sequences is expected to produce a more finer resolution in lineage assignment we, observed low sensitivity in lineage assignment for lineages B, D, J, M and O using whole genome sequences (L, M and S segments). This could be due to few number of whole genome sequences belonging to these lineages. For these lineages we found only single isolates with complete segment sequences available in the current NCBI database. The limited number of sequences belonging to these lineages made it difficult to identify unique lineage defining SNPs with strong statistical power to distinguish lineages. However, despite the limited number of sequences for these lineages, lineage assignment using the glycoprotein gene (Gn) sequence produced accurate and optimal assignment for all the sequences with respect to the Grobbelaar et al. [13] classification of 15 lineages. Furthermore, despite low accuracy in the assignment of sequences belonging to lineage G and J using whole genome sequences, the glycoprotein lineage assignment classifier correctly assigned G (HM587087, HM587083, AF134499 and DQ380218) and J (DQ380222) sequences but with low bootstrap support values ranging between 61 and 64. Although the Gn classifier performs better in these two lineages, a robust bootstrapping (by increasing the number of replications) can be undertaken to ensure that a desired bootstrap threshold value is achieved.

Testing the complete M-segment sequences using the glycoprotein (Gn) gene classifier, lineage assignment scored 100% sensitivity, specificity and accuracy. However, sequence accessions DQ380216, DQ380215 (lineage G) and DQ380222 (lineage J) were assigned with low bootstrap support values of 67, 68 and 35 respectively. A detailed classification performance can be found in Table 6. Generally, using the glycoprotein gene (Gn) as a classifier with the complete M-segment sequences as input, produced complete lineage assignments. Overall, the Gn classifier was able to produce 100% assignment (with respect to the 15 lineage classification produced by Grobbelaar et al) across all the lineages with no false positives identified.

**Fig. 4:**
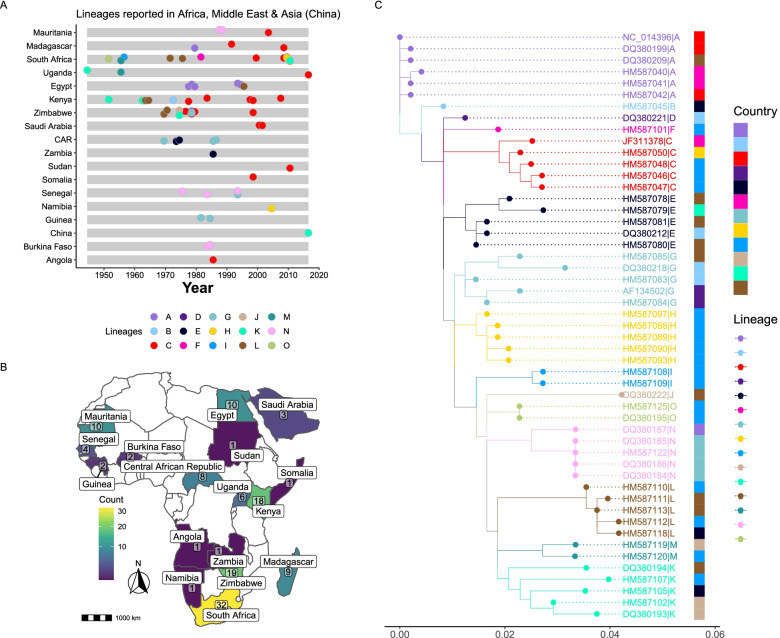
Distribution of RVFV lineages in Africa and Middle East. **A** Lineages reported in Africa and the Middle East (Saudi Arabia) sampled between 1944 to 2016. **B** Map of Africa and Saudi Arabia indicating the number RVFV sequences for the M-segment (partial and complete) as of 28th May 2021 for the 129 sequences used in the lineage assignment. **C** Maximum likelihood phylogenetic tree using glycoprotein (Gn) representative sequences (*n* = 51) showing geographical distribution of lineages. The tips of the tree are colored according to their country of origin. CAR, Central African Republic

The maximum likelihood phylogenetic reference trees that we generated were able to resolve the 15 lineages (A-O) with bootstrap support values of over 70%. The reference trees generated using both the glycoprotein (Gn) gene and whole genome sequences (L, M and S-segments) had similar topologies (Fig. 3). This congruence is indicative of low occurrence of reassortment of the Rift Valley fever virus [11]. From the phylogenetic lineage analyses, there is no clear pattern in lineages occurrence in Africa. This may indicate widespread transmission and dispersal of the virus across the African continent. Most countries that have experienced RVF outbreak have reported more than a single circulating lineage. However, since it was first reported in 1976, lineage C continues to be the most predominant lineage in Africa (Fig. 4).

In addition to sequences retrieved from NCBI database, we also evaluated the tool using whole RVFV genome sequences that we generated from clinical livestock samples of a recent outbreak in Kenya. The sequence data was generated using Illumina technologies. These technologies have been used to conduct genomic epidemiology of pathogens at varying scales of outbreaks [35]. Lineage analysis of the outbreak samples using both Gn and whole genome classifiers showed that the isolates belong to lineage C. This assignment was supported by maximum likelihood phylogenetic analysis that produced a monophyletic clustering for the 5 samples with high (> 90%) bootstrap values.

## Conclusion

We have developed RVFV typing tool with both command line and user-friendly web-based interface usability. RVFV Typing tool presented here allows for fast and accurate classification of RVFV species and lineages within a few minutes. Lineages can be confidently assigned using the whole genome (L, M, & S-segments) and/or the partial glycoprotein Gn (490 bp) sequences. Based on the 15 lineages proposed by Grobbelaar,the glycoprotein (Gn) gene classifier showed consistency in lineage assignment of the partial Gn and whole genome sequence of the M segment. In resource limited settings where whole genome sequences may not be readily generated, partial sequences of the M segment can be used for typing. In addition, the Gn classifier can still accurately assign lineages with samples where full length genome segments are provided as input. Although our analysis used the current 15 lineages produced by Grobbelaar et al. as reference to assess the performance of the classifiying tool, further analysis using whole genome sequence as classifier should provide a finer and higher resolution on lineage assignment with coverage of the entire genome, providing comprehensive information that may include possible genetic reassortments.

## Supplementary Information

**Additional file 1.**

## Data Availability

Provisional accession numbers of the sequences are OM744365 - OM744379 on NCBI. Raw sequence reads in fastq format are provisionally available on the Sequence Read Archive with the accession PRJNA811331.
