# Supplementary material for: Genomic surveillance of Rift Valley fever virus: from sequencing to lineage assignment
Source: BMC Genomics. 2022 Jul 18;23:520. doi: 10.1186/s12864-022-08764-6 (PMC9295512; doi:10.1186/s12864-022-08764-6)
Supplement: Supplementary file 1 — Additional file 1. [file 12864_2022_8764_MOESM1_ESM.docx]

**Supplementary materials**

*Sample Collection and Processing*

During the month of July 2021, we responded to suspect livestock RVF outbreak reports in Kiambu county in central Kenya (Figure S1). Clinical investigation of the affected cattle revealed fever (body temperature above 40°C), lack of appetite, abortions and diarrhea that are typical of Rift Valley fever infection. In one farm, there were reports of abortions in 7 cattle and 2 mortalities. The area where adult cattle were raised was swampy while calves were secluded in a separate dry area. Whole blood samples were collected from livestock in plain vacutainer and EDTA tubes respectively. These were stored in cool box after which serum was obtained from the plain vacutainer tubes through centrifugation at 12000xg for 15 minutes. Samples were immediately transported to the International Livestock Research Institute (ILRI) laboratories for downstream analyses.

*IgM antibody capture ELISA*

All the Rift Valley fever suspected samples were first screened using enzyme linked immunosorbent assay (ELISA) for the detection of anti-nucleoprotein IgM antibodies in sera. This was done using the IDVet -ELISA kits as per the manufacturer’s instructions (IDVet Innovative Diagnostic, Grabels, France). Briefly, serum samples in a 1:10 dilution were added in plates coated with anti-bovine-ovine-caprine IgM polyclonal antibody. Plates were incubated for 1 hour at 37°C, washed and RVF nucleoprotein added and incubated. This was followed by 3 washes, addition of anti-NP-HRP conjugate, and an incubation. A substrate buffer containing 0.1mg/mL 3,3’,5,5-tetramethylbenzidine (TMB) (Thermo Scientific, Rockford, IL) was then added after the final wash and optical density (OD) values recorded using an ELISA reader. The presence of IgM antibodies to RVFV was detected by a blue coloration which changed to yellow following the addition of the stop solution. The contents of the wells of the microplate were analyzed at 450nm by the ELISA reader. For each IgM antibody capture ELISA experiment internal controls were included. The optical densities at 450 nm obtained from the samples were validated in accordance with the manufacturer’s instructions as indicated below:

$$net OD =OD_{even well} -OD_{odd well}$$

The plate was valid if the mean value of the net positive control $OD >0.35$ and the ratio of the mean values of the net positive and negative control (absolute value of ODs) > 3 (${OD_{positive control}}/{net}OD_{negative control >3}$)

For each sample, the percentage of the ratio of sample and positive control (s/p*100%) was calculated as:

$$S/P\% =net{OD_{sample}}/{net}OD_{positive control}*100$$

Sample presenting a S/P percentage (S/P%):

1. $\leq40\% is negative$
2. $40\%-50\% is Doubtful$
3. $\geq50\% is Positive$

*Virus Isolation in culture*

Virus isolation was performed in containment Biosafety Level 3 (BSL3) facility at ILRI. Vero cells (Gibco-Biocult) were cultured in T25 flasks in growth Media (Minimum essential media, Eagles, Sigma with Earle’s salts and reduced NaHCO_3_) supplemented with 10% heat inactivated Fetal Bovine Serum (FBS), 2% L-glutamine and 2% penicillin/amphotericin and incubated at 37°C in 5% CO_2_. The T25 flasks with confluent monolayers were then inoculated with 200µL serum. Prior to inoculation, the growth media was poured out and after inoculation, the flasks were incubated for 1 hour at 37^o^C to allow for virus adsorption. Up to 5ml of maintenance media (Minimum essential media, Eagles, Sigma with Earle’s salts and reduced NaHCO_3_) supplemented with 2% heat inactivated Fetal Bovine Serum (FBS), 2% L-glutamine and 2% penicillin/amphotericin was then added to each flask and incubated at 37°C and 5% CO_2_. Flasks were observed daily for a period of 2 weeks using an inverted microscope for evidence of cytopathic effects (CPE). Flasks showing evidence of CPE were freeze/thawed and cell culture supernatant filtered, harvested, and stored at -80^o^C for viral RNA isolation.

Viral RNA was isolated from 140µL of the filtered cell culture supernatant using QIAmp Viral RNA kit (QIAGEN, Hilden, Germany), according to the manufacturer’s instructions. Cycle threshold (Ct) values were determined for the RNA samples using probe-based reverse transcription quantitative real-time PCR against a highly conserved domain located on the L-segment of the virus for RVFV detection (using 5’ Fam reporter dye and 3’ BHQ1 quencher dye). The following primers and probe set were used: RVFL-2912fwdGG (5′-TGAAAATTCCTGAGACACATGG-3′), RVFL-2981revAC (5′-ACTTCCTTGCATCATCTGATG-3′), and RVFL-probe-2950 (5′-CAATGTAAGGGGCCTGTGTGGACTTGTG-3′) [1]. One-Step Probe RT-qPCR ReadyMix in a reaction of 10 µl using a final concentration of 0.3 µM for primers and 0.1 µM for probe in a PCR System (Applied Biosystems) installed at the Real-Time PCR Platform of ILRI. The reaction was carried in a series of incubation steps as follows: 50°C for 10 minutes, 95°C for 1 minute, 95°C for 3 seconds for 40 cycles and 60°C for 30 minutes.

***Library prep***

Sequencing libraries were prepared using the total RNA NEBNext Ultra II Library Prep Kit for Illumina (New England Biolabs, UK) following kit manufacture’s instruction. In brief, depletion of ribosomal RNA was performed by hybridization of the depletion probe to 12 µL of sample RNA in a 15 µL reaction. Both RNAse H and DNase I digestion were performed to prevent degradation. RNA samples were purified with NEBNext RNA sample purification beads. Ribosomal RNA depleted total RNA was eluted in 5 µL nuclease-free water followed by fragmentation and priming using random primers in a 10 µL reaction at 94°C for 8 minutes. First strand cDNA synthesis was performed using the fragmented and primed sample by mixing with NEBNext First Strand Synthesis Enzyme mix. The reaction was carried in a series of incubation steps as follows: 25°C for 10 minutes, 42°C for 15 minutes, 70°C for 15 minutes and holding at 4°C. After first strand cDNA synthesis, second strand cDNA synthesis was immediately carried out by directly mixing the first strand cDNA synthesis product with the second strand synthesis buffer and the enzyme mix following the Kit manufacturer’s instructions. The double-stranded cDNA product was purified using NEBNext sample purification beads. End repair and adaptor ligation was performed using kit’s reagents. Adaptor-ligated product was cleaned using sample purification beads followed by library PCR enrichment using the NEBNext Ultra II Q5 Master mix and premixed NEBNext oligos in a 50µL reaction for 15 cycles. PCR-enriched libraries were cleaned using sample purification beads in a 1:0.9 ratio. We assessed the quality of our libraries through gel electrophoresis (1% agarose gel in 1X TAE buffer). We quantified our libraries using KAPA Library quantification kit (Roche, Boston, USA) and pooled our samples at 4nM before denaturing and loading at 1.5 pM for sequencing. The pooled library was loaded on the Illumina NextSeq 550 for a paired-end 2 × 150 bp sequencing with the Mid Output cartridge. The run took <26 hours to obtain 47.7Gb of compressed sequencing data.

***Virus genome assembly***

Consensus genome sequences for each barcoded sample was generated using a nextflow pipeline we developed (<https://github.com/ajodeh-juma/viclara>). In brief, the demultiplexed FASTQ files were concatenated/merged for each lane for both forward and reverse reads. The raw data was checked for quality metrics using FastQC v0.11.9 [2]. Low quality reads and adaptors were trimmed using fastp [3]. Alignment to the GenBank reference genome was performed using Burrows-Wheeler Alignment (BWA) [4]. Alignment metrics were generated with SAMtools [5] and only samples surpassing a minimum mapping threshold of 500 were kept in subsequent downstream analysis. Using each segment’s reference (ZH-548 strain) and genome annotation file, variants were called on the pileup generated from the marked duplicates alignments. Only positions with ≥ 10X genome coverage and with ≥ 20 base qualities were used to produce consensus alleles. Regions with lower coverage and base quality were masked with N characters. Genome wide coverage was computed using BEDTools [6].

**Supplementary Tables**

**Table S1: RVFV Lineage defining single nucleotide polymorphisms (SNPs) for complete S-segment.** For each lineage sequences, SNPs were identified in comparison to the reference (NC_014395).

| Lineage | SNPs | Total |
| --- | --- | --- |
| A | 1233CT;1325AG | 2 |
| B | 40TC;427TC;538TC;684TC;724CT;758AG;796GA;893CT;945GA;978GA;990TC;1089TC;1194AG;1233CT;1325AG;1451TG | 16 |
| C | 101TA;109AT;427TC;478TC;487CT;534CT;538TC;544CT;586TC;616CT;625TC;631AG;684TC;758AG;945GA;990TC;996GA;1089TC;1180CT;1194AG;1325AG;1451TG;1545TC | 23 |
| D | 100CT;101TA;109AT;169CT;233TC;283GA;415TA;427TC;439CA;451TC;478TC;534CT;538TC;574GA;586TC;616CT;625TC;631AG;684TC;724CT;758AG;784GA;796GA;884TC;893CT;945GA;978GA;990TC;996GA;1089TC;1128GA;1152AG;1180CT;1194AG;1233CT;1299CT;1325AG;1449CT;1451TG;1545TC | 40 |
| E | 101TA;109AT;115TC;283GA;427TC;478TC;487CT;534CT;538TC;544CT;586TC;616CT;619TA;625TC;631AG;684TC;724CT;758AG;796GA;823GA;884TC;892TA;893CT;945GA;978GA;990TC;996GA;1089TC;1098AG;1180CT;1194AG;1233CT;1254GA;1301AG;1325AG;1451TG | 36 |
| F | 101TA;109AT;283GA;534CT;538TC;616CT;625TC;631AG;684TC;724CT;758AG;945GA;990TC;996GA;1089TC;1180CT;1194AG;1233CT;1325AG;1451TG;1545TC | 21 |
| G | 101TA;109AT;283GA;392CT;406CT;409AT;427TC;439CT;478TC;487CT;534CT;538TC;544CT;586TC;616CT;625TC;631AG;673AT;675CG;684TC;724CT;758AG;796GA;823GA;884TC;890CT;893CT;945GA;952AT;966CT;978GA;990TC;996GA;1002CT;1089TC;1098AG;1180CT;1194AG;1233CT;1325AG;1362GA;1428CT;1451TG;1545TC;1548AG | 45 |
| H | 101TA;214CT;283GA;310AG;427TC;442TC;445AG;478TC;534CT;538TC;607CT;616CT;620CA;625TC;684TC;724CT;758AG;760TC;787AT;796GA;814AG;863AG;893CT;942AG;945GA;978GA;990TC;996GA;1029TA;1089TC;1180CT;1185TC;1194AG;1215GA;1233CT;1299CT;1325AG;1425TC;1451TG;1530TC;1539GA | 41 |
| I | 4CT;101TA;109AT;115TC;208TC;214CT;229CT;283GA;310AG;394GA;427TC;478TC;526GA;534CT;538TC;544CT;586TC;607CT;610AT;616CT;625TC;631AG;649TC;664TC;679CT;684TC;700CT;724CT;758AG;796GA;839AG;884TC;885NA;893CT;902CA;936AC;945GA;960AC;966CT;990TC;996GA;1077AG;1089TC;1119GA;1158TC;1180CT;1194AG;1212GA;1233CT;1254GA;1325AG;1329GA;1374TC;1451TG;1512AT;1545TC;1560CT | 57 |
| J | 101TA;109AT;199AG;283GA;361AG;406CT;427TC;478TC;487CT;490CT;493TC;534CT;538TC;544CT;586TC;589CA;610AT;616CT;625TC;630AG;631AG;684TC;700CT;724CT;758AG;796GA;864AG;884TC;885NA;887CT;893CT;902CA;936AC;945GA;960AC;975AG;987AG;990TC;996GA;1014CT;1065CT;1089TC;1125GA;1158TC;1180CT;1194AG;1203GA;1233CT;1254GA;1290CT;1325AG;1344TC;1350CT;1374TC;1380TC;1401AG;1451TG;1482AG;1542AG;1545TC;1560CT;1563TC | 62 |
| K | 101TA;109AT;283GA;427TC;502GA;534CT;538TC;547CT;586TC;610AC;616CT;631AG;684TC;724CT;758AG;884TC;936AC;945GA;978GA;990TC;996GA;1089TC;1158TC;1180CT;1194AG;1233CT;1254GA;1325AG;1374TC;1428CT;1451TG;1545TC | 32 |
| L | 101TA;109AT;283GA;478TC;487CT;534CT;544CT;610AT;616CT;625TC;631AG;684TC;724CT;758AG;796GA;885NA;893CT;902CA;936AC;945GA;990TC;996GA;1089TC;1158TC;1180CT;1194AG;1233CT;1254GA;1325AG;1374TC;1451TG;1545TC;1560CT | 33 |
| M | 101TA;109AT;283GA;310AG;319TC;385AG;391AC;427TC;478TC;487CT;502GA;518TC;520AG;534CT;538TC;544CT;547CT;586TC;593TC;610AC;616CT;625TC;631AG;684TC;724CT;758AG;796GA;838AG;869GA;872CT;875AT;884TC;885NA;886NC;893CT;902CA;936AC;945GA;960AC;972CT;978GA;990TC;996GA;1032AG;1083CT;1089TC;1158TC;1167CT;1180CT;1233CT;1254GA;1301AG;1325AG;1329GA;1374TC;1386AG;1428CT;1451TG;1536TC;1545TC;1551GA;1560CT | 62 |
| N | 101TA;109AT;232AC;283GA;409AG;415TC;427TC;451TC;534CT;538TC;544CT;586TC;607CT;610AT;616CT;625TC;631AG;684TC;721TC;724CT;758AG;787AT;796GA;884TC;885NA;893CT;902CA;916AG;936AC;945GA;960AC;990TC;996GA;1089TC;1158TC;1180CT;1194AG;1233CT;1254GA;1325AG;1340GT;1374TC;1416TC;1451TG;1458GA;1545TC;1560CT;1596CT | 48 |
| O | 42AT;85TC;101TA;109AT;226AG;283GA;382AG;427TC;478TC;487CT;534CT;538TC;544CT;586TC;610AT;616CT;625TC;631AG;649TC;684TC;724CT;742CT;758AG;796GA;884TC;885NA;893CT;902CA;936AC;945GA;960AC;978GA;990TC;996GA;999TC;1089TC;1152AG;1158TC;1180CT;1194AG;1233CT;1254GA;1325AG;1332AG;1374TC;1451TG;1542AG;1545TC;1560CT | 49 |

**Table S2: RVFV Lineage defining single nucleotide polymorphisms (SNPs) for complete M-segment.** For each lineage sequences, SNPs were identified in comparison to the reference (NC_014396).

| Lineage | SNPs | Total |
| --- | --- | --- |
| A | 715TA;1717AG | 2 |
| B | 269TC;614AG;715TA;830GA;1103TC;1142TC;1526AT;1616TC;1717AG;1841TC;1946CT;2061TC;2064TC;2354CT;2471AG;2682GA;3195TA;3368CT;3628AG;3704AG;3821AG | 21 |
| C | 110CT;179GA;194TC;212AG;303GA;323CT;635GA;715TA;731CT;737CT;767CT;836TA;926GA;1103TC;1163CT;1190TC;1241AG;1355AT;1526AT;1577CT;1580CT;1616TC;1787GA;1817TC;1841TC;1850GA;1911AG;1913AG;1931CT;2057GA;2061TC;2064TC;2147TC;2303TC;2327TC;2330AG;2334TC;2354CT;2369CT;2441GA;2471AG;2735CT;2885AG;3020AG;3053AG;3101AG;3122AG;3195TA;3236CT;3239TC;3254AG;3263CT;3347GA;3353GA;3368CT;3374TC;3628AG;3640TA | 58 |
| D | 56GA;110CT;125TC;158AG;179GA;212AG;239AG;323CT;396AG;614AG;715TA;731CT;839TC;926GA;1103TC;1142TC;1163CT;1195GA;1325AG;1337GT;1526AT;1577CT;1616TC;1717AG;1841TC;1850GA;1859CT;1892CT;1911AG;1913AG;1931CT;1946CT;1997TC;2057GA;2061TC;2064TC;2135TC;2186AG;2285AG;2303TC;2327TC;2334TC;2354CT;2360CT;2363GA;2366AG;2369CT;2443CT;2471AG;2918AG;3098CT;3195TA;3236CT;3263CT;3290GA;3353GA;3368CT;3512CT;3596CT;3621AG;3628AG;3640TA;3676GA;3681GT;3691GA;3704AG;3720AT;3740AT;3813GA;3821AG;3824CT;3842CT | 72 |
| E | 125TC;179GA;323CT;614AG;626TC;715TA;731CT;854TA;926GA;1103TC;1142TC;1151TC;1163CT;1166AG;1316CT;1526AT;1616TC;1717AG;1793AG;1841TC;1850GA;1892CT;1911AG;1913AG;1946CT;2024TC;2036CT;2057GA;2061TC;2064TC;2135TC;2147TC;2186AG;2214TC;2237AC;2285AG;2303TC;2334TC;2354CT;2369CT;2471AG;2486GA;2600TA;2643TC;2805TC;2903TC;2921AG;3017TC;3195TA;3236CT;3263CT;3266GA;3269TC;3290GA;3296AG;3326TC;3368CT;3551TC;3596CT;3628AG;3640TA;3641GA;3676GA;3704AG;3745TC;3824CT | 66 |
| F | 110CT;125TC;179GA;212AG;323CT;497AG;614AG;659AG;662GA;677CT;715TA;731CT;816AG;902GA;926GA;1079GA;1103TC;1106GA;1142TC;1163CT;1253GA;1319TC;1337GT;1454TC;1526AT;1577CT;1616TC;1649CT;1691TC;1717AG;1813TC;1841TC;1850GA;1868AG;1874GA;1892CT;1911AG;1913AG;1931CT;1946CT;1985AG;2057GA;2061TC;2064TC;2135TC;2186AG;2216GA;2249GA;2255AG;2285AG;2303TC;2327TC;2334TC;2354CT;2360CT;2363GA;2369CT;2471AG;2495TC;2534TC;2599CG;2708CT;2822CT;2936CT;2981CA;3011AG;3152AG;3155GA;3195TA;3230TC;3236CT;3263CT;3290GA;3353GA;3368CT;3596CT;3628AG;3640TA;3645TC;3650AG;3676GA;3704AG;3720AT;3721AG;3733AT;3740AT;3824CT | 87 |
| G | 110CT;125TC;179GA;212AG;323CT;344CT;608AG;614AG;620TC;715TA;731CT;920AG;926GA;1085TC;1103TC;1142TC;1163CT;1171CA;1173AT;1406GA;1490AG;1526AT;1616TC;1622GA;1715TC;1717AG;1841TC;1850GA;1892CT;1911AG;1913AG;1918AG;1946CT;1967AG;2029CT;2030CT;2036CT;2057GA;2061TC;2064TC;2067TC;2126CT;2135TC;2147TC;2186AG;2285AG;2303TC;2327TC;2334TC;2354CT;2369CT;2432GA;2471AG;2486GA;2603AG;2855AG;2933TC;2945CA;2993AT;3041AG;3195TA;3236CT;3263CT;3290GA;3344GA;3352AG;3359CT;3368CT;3513CT;3551TC;3596CT;3628AG;3640TA;3676GA;3706TC;3725AC;3732AG;3821AG;3824CT | 79 |
| H | 92CT;125TC;179GA;212AG;323CT;515GA;600TC;614AG;623CT;626TC;635GA;715TA;731CT;770TC;920AG;926GA;1103TC;1142TC;1157AG;1163CT;1169AT;2303TC;3628AG | 23 |
| I | 33TC;110CT;125TC;179GA;212AG;236GA;239AG;305TC;323CT;386TC;515GC;557CT;605TC;635GT;715TA;731CT;752CT;794TC;806TC;833CT;920AG;980GA;986CT;998TC;1049GA;1103TC;1115GA;1142TC;1163CT;1304GA;1343GA;1349GA;1427TA;1466TC;1481GA;1493GA;1520AC;1526AT;1553CT;1616TC;1691TC;1717AG;1790TC;1799TC;1803AG;1823TC;1834GA;1841TC;1850GA;1853GA;1892CT;1895AT;1911AG;1913AG;1946CT;1996TC;2001GA;2030CT;2036CT;2057GA;2061TC;2064TC;2135TC;2141GA;2147TC;2162AG;2180GA;2186AG;2240TC;2255AG;2285AG;2303TC;2330AG;2334TC;2354CT;2369CT;2468GA;2471AG;2489TC;2531GA;2555GA;2582CT;2603AG;2633AT;2678TG;2831GA;2834CT;2906AG;3005AG;3008TC;3011AG;3089GA;3101AG;3195TA;3197TC;3213AG;3236CT;3263CT;3290GA;3296AG;3305GA;3359CT;3368CT;3437TC;3548CT;3572CT;3596CT;3621AG;3628AG;3638GA;3640TA;3659CT;3664CG;3676GA;3679TC;3681GA;3695AG;3697TC;3702GA;3704AG;3714CT;3725AT;3761TC;3797GA;3798CT;3815TC;3821AG;3824CT | 128 |
| J | 33TC;110CT;125TC;131AC;158AG;179GA;212AG;236GA;239AG;305TC;323CT;350GA;386TC;557CT;605TC;614AG;620TC;626TC;677CT;695TC;715TA;731CT;749AG;785GA;800CT;836TC;860CT;920AG;926GA;953AG;995GA;1007CA;1055TC;1115GA;1142TC;1154GA;1160GA;1161TC;1163CT;1190TC;1250TC;1343GA;1361AG;1412GA;1478TC;1491TC;1526AT;1577CT;1604GA;1610CT;1616TC;1652TC;1691TC;1717AG;1790TC;1793AG;1799TC;1803AG;1820AG;1823TC;1834GA;1841TC;1850GA;1864GA;1880AG;1892CT;1895AT;1911AG;1943CT;1946CT;1949TA;1996TC;2027TC;2030CT;2042TC;2057GA;2061TC;2064TC;2067TC;2084AG;2117CT;2120AG;2123CT;2135TC;2141GA;2147TC;2162AG;2180GA;2183CT;2186AG;2222GA;2240TC;2255AG;2285AG;2303TC;2306TC;2327TC;2334TC;2354CT;2369CT;2384TC;2385CT;2471AG;2486GA;2534TC;2582CT;2732TA;2805TC;2825TC;2831GA;2834CT;2894CT;2906AG;2975GA;2987CT;3011AG;3041AG;3101AG;3195TA;3212TC;3236CT;3239TC;3248GA;3263CT;3296AG;3342CT;3347GA;3401GA;3458TC;3494AG;3497TC;3509TA;3548CT;3572CT;3596CT;3608CT;3621AG;3628AG;3638GA;3640TA;3658GA;3665TC;3669TC;3676GA;3691GA;3697TC;3703GA;3704AG;3714CT;3792CA;3797GA;3819GA;3821AG;3824CT;3829TC;3840AG | 156 |
| K | 33TC;104GA;110CT;125TC;179GA;197TC;236GA;488CT;605TC;626TC;715TA;894CT;1091TC;1103TC;1115GA;1142TC;1250TC;1349GA;1364AC;1526AT;1571TC;1577CT;1616TC;1649CT;1691TC;1707TC;1717AG;1740CT;1790TC;1799TC;1820AG;1823TC;1834GA;1841TC;1850GA;1895AT;1911AG;1913AG;1946CT;1949TC;1988TC;2036CT;2057GA;2061TC;2064TC;2135TC;2141GA;2147TC;2162AG;2180GA;2186AG;2222GA;2240TC;2255AG;2285AG;2303TC;2327TC;2334TC;2354CT;2369CT;2417GA;2471AG;2486GA;2579CT;2582CT;2810GA;2831GA;2834CT;2906AG;3011AG;3101AG;3143TC;3161GA;3195TA;3236CT;3263CT;3290GA;3296AG;3299AG;3392GA;3467CT;3494AG;3506CA;3572CT;3605GA;3638GA;3640TA;3651GA;3663CT;3676GA;3697TC;3704AG;3714CT;3721AT;3727GA;3733AG;3797GA;3804AG;3821AG;3824CT | 100 |
| L | 48TA;89CT;125TC;179GA;197TC;212AG;221GA;236GA;242CT;314CT;323CT;386TC;401GA;488CT;581AT;605TC;626TC;641TC;647TC;653TC;662GA;715TA;731CT;743AG;842GA;866CT;872TC;917CT;920AG;926GA;1061GA;1103TC;1115GA;1122CT;1124AG;1142TC;1163CT;1190TC;1250TC;1274AT;1304GA;1316CT;1343GA;1349GA;1364AC;1385AG;1403AG;1430CT;1511GT;1526AT;1556CT;1571TC;1577CT;1616TC;1649CT;1700CT;1703GA;1707TC;1717AG;1790TC;1796CT;1799TC;1803AG;1805TC;1820AG;1823TC;1834GA;1835GA;1838GT;1841TC;1850GA;1892CT;1895AT;1898CT;1911AG;1913AG;1946CT;1949TC;1955AG;1988TC;1992TC;1996TC;2030CT;2036CT;2057GA;2061TC;2064TC;2135TC;2141GA;2147TC;2162AG;2180GA;2186AG;2222GA;2240TC;2252CT;2255AG;2285AG;2303TC;2334TC;2354CT;2369CT;2396GA;2417GA;2450AG;2471AG;2486GA;2546TC;2570TC;2582CT;2609CT;2612AT;2643TC;2723TC;2831GA;2834CT;2882CT;2906AG;2957TC;3011AG;3044AG;3056TA;3101AG;3143TC;3161GA;3195TA;3215GA;3236CT;3263CT;3290GA;3296AG;3356GA;3368CT;3392GA;3494AG;3521TA;3599TC;3605GA;3628AG;3640TA;3651GA;3663CT;3676GA;3689AG;3697TC;3704AG;3714CT;3721AT;3727GA;3733AG;3786CT;3797GA;3799TA;3804AG;3821AG;3824CT | 156 |
| M | 33TC;54CT;110CT;125TC;149TG;161CT;179GA;182GA;236GA;323CT;386TC;488CT;500CT;557CT;626TC;659AG;671CT;715TA;731CT;857GA;894CT;920AG;924TC;926GA;992GT;1034TC;1103TC;1115GA;1142TC;1151TC;1163CT;1195GA;1214TC;1250TC;1304GA;1307CT;1322TC;1337GA;1343GA;1349GA;1364AC;1526AT;1571TC;1577CT;1616TC;1649CT;1667CT;1707TC;1717AG;1790TC;1799TC;1803AG;1820AG;1823TC;1834GA;1841TC;1850GA;1871TA;1892CT;1895AT;1911AG;1913AG;1946CT;1949TC;1996TC;2030CT;2036CT;2057GA;2061TC;2064TC;2082GA;2135TC;2141GA;2147TC;2153CT;2162AG;2180GA;2186AG;2222GA;2240TC;2255AG;2285AG;2303TC;2315GA;2327TC;2334TC;2354CT;2369CT;2417GA;2444CT;2459GA;2471AG;2486GA;2582CT;2609CT;2831GA;2834CT;2906AG;2963GA;3011AG;3050TC;3092AG;3101AG;3143TC;3161GA;3191TC;3195TA;3236CT;3263CT;3290GA;3296AG;3299AG;3368CT;3437TC;3488TC;3494AG;3506CT;3521TA;3548CT;3572CT;3605GA;3625GC;3628AG;3638GA;3640TA;3651GA;3654AG;3663CT;3676GA;3689AG;3697TC;3704AG;3714CT;3720AC;3721AT;3727GA;3734TC;3754TC;3797GA;3804AG;3821AG;3824CT;3841GA | 143 |
| N | 33TC;110CT;125TC;179GA;194TC;212AG;236GA;245GA;323CT;326CT;386TC;500CT;557CT;605TC;620TC;626TC;671CT;704CT;715TA;734CT;794TC;821TC;920AG;926GA;1025AG;1103TC;1112GA;1115GA;1142TC;1163CT;1187GA;1250TC;1304GA;1343GA;1364AC;1433GA;1463TC;1526AT;1535TA;1553CT;1577CT;1586TC;1616TC;1707TC;1717AG;1742GA;1790TC;1799TC;1803AG;1823TC;1834GA;1841TC;1850GA;1874GA;1892CT;1895AT;1911AG;1913AG;1946CT;1982AG;1996TC;2036CT;2061TC;2064TC;2123CA;2141GA;2147TC;2162AG;2180GA;2186AG;2213TC;2222GA;2240TC;2246CA;2285AG;2294GA;2303TC;2315GA;2324CT;2327TC;2334TC;2354CT;2369CT;2444CT;2459GA;2471AG;2486GA;2495TC;2519AG;2582CT;2624CT;2789TC;2831GA;2834CT;2865AC;2879TC;2903TC;2906AG;2966AT;2981CT;3011AG;3051CT;3101AG;3126TC;3137CT;3161GA;3195TA;3236CT;3263CT;3290GA;3296AG;3494AG;3548CT;3572CT;3596CT;3614GA;3620TC;3628AG;3640TA;3648GA;3676GA;3690GA;3692TC;3697TC;3704AG;3714CT;3753GA;3755TC;3756GA;3794AG;3797GT;3821AG;3824CT | 133 |
| O | 33TC;110CT;125TC;179GA;212AG;236GA;320TC;323CT;350GA;386TC;515GA;557CT;605TC;629CT;715TA;731CT;806TC;920AG;926GA;1103TC;1106GA;1115GA;1142TC;1163CT;1205AG;1243AG;1250TC;1304GA;1343GA;1394GA;1415GA;1526AT;1571TC;1577CT;1580CA;1598TC;1616TC;1622GA;1691TC;1697GA;1717AG;1790TC;1799TC;1803AG;1814TC;1823TC;1834GA;1841TC;1850GA;1892CT;1895AT;1911AG;1913AG;1946CT;1967AG;1996TC;2006CT;2030CT;2036CT;2057GA;2061TC;2064TC;2093TC;2141GA;2147TC;2162AG;2180GA;2186AG;2240TC;2255AG;2285AG;2303TC;2327TC;2330AG;2334TC;2354CT;2369CT;2399CT;2453GA;2471AG;2486GA;2582CT;2603AG;2607AG;2615CT;2699CT;2831GA;2834CT;2840CT;2849TC;2852TC;2906AG;2960TC;3008TC;3011AG;3101AG;3113CT;3134TG;3195TA;3197TC;3236CT;3263CT;3290GT;3305GA;3368CT;3548CT;3572CT;3575CT;3596CT;3616TC;3628AG;3640TA;3666AG;3676GA;3692TC;3693CT;3697TC;3703GA;3704AG;3714CT;3725AT;3797GA;3821AG;3824CT;3845GA;3846GA;3866AG | 127 |

**Table S3: RVFV Lineage defining single nucleotide polymorphisms (SNPs) for complete L-segment.** For each lineage sequences, SNPs were identified in comparison to the reference (NC_014397).

| Lineage | SNPs | Total |
| --- | --- | --- |
| A | 822AC;1057CT;1427GA;3768TC;6303GA | 5 |
| B | 86TA;208CT;822AC;851GA;1020TC;1057CT;1227CT;1353CT;1427GA;1605AG;1710TC;2005GA;2112TC;2178TC;2421TG;2487GA;2943CT;3072GA;3090CT;3210CT;3348GA;3489TC;3642TA;3723AG;3768TC;3906CT;4108TC;5070CT;5079AG;5157CT;5175TC;5239TC;5406GA;5490TC;5968GA;6255AG;6303GA | 37 |
| C | 198CT;207GA;366TC;519TC;544CT;744CT;753AG;822AC;851GA;855CT;876TC;879GA;881CT;922GA;1020TC;1026CT;1057CT;1065GA;1090TC;1119CT;1227CT;1290TC;1427GA;1500TC;1509GA;1524CT;1533CT;1695TC;1830CT;1932TC;2005GA;2106TC;2208GA;2331GA;2406CT;2421TG;2424CT;2448CT;2520GA;2529CT;2571GA;2598TC;2628AG;2637AG;2943CT;3042GA;3072GA;3108AG;3210CT;3283CT;3291TC;3318CT;3348GA;3426TC;3465TC;3642TA;3675AG;3768TC;3819GA;3835CT;3894AG;4015GA;4108TC;4110GA;4272TC;4557TC;4584GA;4689AG;4812TC;4815AT;4884TC;4887TC;4953AG;4968AG;5061AT;5067CA;5070CT;5079AG;5157CT;5187GA;5217TC;5349AG;5406GA;5433CT;5596CT;5664GA;5730CT;5784GA;5916AG;5934GA;5955CT;5970CT;5994CT;6051TC;6303GA | 95 |
| D | 86TA;450CT;516TC;519TC;544CT;666GA;699CT;744CT;822AC;851GA;922GA;930AG;1017TC;1020TC;1057CT;1090TC;1119CT;1161AG;1227CT;1350CT;1353CT;1401GA;1427GA;1497GA;1500TC;1533CT;1588CT;1605AG;1626CT;1695TC;1710TC;1794TC;2005GA;2025TC;2136CT;2211TA;2274CT;2280CT;2358CT;2385GA;2421TG;2424CT;2463TC;2475TC;2520GA;2628AG;2637AG;2751CT;2805TC;2838GA;2853AT;2886TC;2943CT;3042GA;3072GA;3090CT;3210CT;3243AG;3250TC;3318CT;3348GA;3426TC;3489TC;3627CT;3642TA;3648CT;3684GA;3723AG;3768TC;3831AG;3894AG;3903TG;3906CT;4015GA;4053CT;4077AG;4086AG;4108TC;4167GA;4272TC;4584GA;4689AG;4815AT;4818AG;4872CT;5002AG;5070CT;5079AG;5157CT;5175TC;5181TC;5187GA;5215AG;5217TC;5253AT;5268CT;5334TC;5349AG;5406GA;5490TC;5604CT;5664GA;5730CT;5766GA;5784GA;5796AG;5850AG;5853CT;5934GA;5968GA;5994CT;6117TC;6147GA;6231AG;6255AG;6303GA | 116 |
| E | 54GA;86TA;132CT;243CT;447AG;453TC;516TC;519TC;544CT;744CT;822AC;851GA;879GA;881CT;922GA;1017TC;1020TC;1057CT;1090TC;1119CT;1188GA;1227CT;1350CT;1353CT;1427GA;1500TC;1533CT;1572CT;1605AG;1626CT;1680TC;1710TC;1830CT;1911TC;2005GA;2106TC;2232CT;2358CT;2364AG;2406CT;2421TG;2424CT;2487GA;2520GA;2569TC;2571GA;2628AG;2637AG;2658AG;2751CT;2895TC;2943CT;3042GA;3072GA;3090CT;3135TC;3210CT;3283CT;3318CT;3348GA;3426TC;3489TC;3642TA;3660AG;3723AG;3768TC;3894AG;3906CT;3996GA;4015GA;4108TC;4207TC;4272TC;4551GA;4554CT;4584GA;4620TC;4689AG;4696TC;4725TC;4731CT;4815AT;4926GA;4953AG;4968AG;5070CT;5079AG;5157CT;5187GA;5217TC;5253AT;5349AG;5406GA;5490TC;5596CT;5730CT;5784GA;5925AG;5934GA;5968GA;5970CT;5994CT;6240GA;6255AG;6276GA;6303GA | 106 |
| F | 86TA;340TC;516TC;519TC;544CT;744CT;822AC;851GA;922GA;1020TC;1057CT;1090TC;1119CT;1227CT;1350CT;1353CT;1427GA;1500TC;1533CT;1605AG;1614CT;1626CT;2005GA;2358CT;2424CT;2487GA;2628AG;2637AG;2751CT;2943CT;3042GA;3072GA;3090CT;3210CT;3348GA;3426TC;3489TC;3642TA;3723AG;3768TC;3894AG;3906CT;4108TC;4272TC;4584GA;4689AG;4815AT;5070CT;5079AG;5187GA;5217TC;5253AT;5349AG;5406GA;5490TC;5730CT;5784GA;5934GA;5940CT;5994CT;6117TC;6303GA | 62 |
| G | 54GA;86TA;285GA;357CA;447AG;468TC;516TC;519TC;544CT;585GA;658CT;744CT;786TC;822AC;851GA;879GA;881CT;922GA;1017TC;1020TC;1057CT;1084TA;1090TC;1119CT;1128GA;1227CT;1249AG;1350CT;1353CT;1427GA;1458GA;1488GA;1500TC;1503CT;1533CT;1551TA;1572CT;1605AG;1626CT;1701TC;1704GA;1710TC;1740TC;1767CT;1770CT;1830CT;1929GA;2005GA;2028TA;2106TC;2232CT;2268AG;2271AT;2358CT;2406CT;2421TG;2424CT;2487GA;2520GA;2569TC;2571GA;2628AG;2637AG;2643CT;2703TC;2751CT;2943CT;3042GA;3072GA;3090CT;3210CT;3291TC;3318CT;3348GA;3426TC;3489TC;3516GA;3642TA;3681CT;3723AG;3744TC;3768TC;3894AG;3895TC;3906CT;4015GA;4053CT;4108TC;4125CT;4272TC;4359TC;4444TC;4551GA;4584GA;4689AG;4731CT;4812TC;4815AT;4833GA;4839TC;4953AG;4968AG;5025GA;5070CT;5079AG;5157CT;5181TC;5187GA;5217TC;5253AT;5307CT;5335AG;5349AG;5406GA;5490TC;5517GA;5596CT;5628TC;5661GA;5664GA;5730CT;5766GA;5784GA;5904TC;5913GA;5934GA;5940CT;5968GA;5994CT;6255AG;6303GA;6304TC;6332TC | 133 |
| H | 86TA;102AG;147AG;150TC;156AT;377TC;465TC;516TC;519TC;540TC;544CT;549GT;637CT;744CT;780TC;798CT;822AC;827AG;846CT;851GA;897AG;900CT;912TC;924AG;981CT;1020TC;1047TC;1057CT;1119CT;1185AG;1188GA;1227CT;1290TC;1353CT;1374AG;1427GA;1456TC;1496GA;1605AG;1620TC;1638AG;1647AG;1671CA;1710TC;1788GA;1863CT;1971AG;1986CT;2005GA;2019AG;2193TC;2217CT;2247GA;2340TC;2358CT;2364AG;2421TG;2424CT;2487GA;2520GA;2526CT;2550TC;2568GA;2637AG;2700AG;2820GA;2907TC;2943CT;2961GA;2979AT;3018TC;3042GA;3072GA;3090CT;3210CT;3231CT;3250TC;3297CT;3318CT;3348GA;3426TC;3468CT;3489TC;3513GA;3642TA;3675AG;3723AG;3732AG;3768TC;3816AG;3891CT;3894AG;3906CT;4108TC;4305AG;4335TC;4374GA;4410TC;4530CT;4560CT;4575GA;4584GA;4662TC;4689AG;4722AG;4755TC;4773AG;4812TA;4833GA;4863CT;4911CT;5016AG;5070CT;5079AG;5082CT;5139CT;5154AG;5157CT;5181TC;5187GA;5253AT;5296GA;5304TC;5406GA;5442AG;5490TC;5538AG;5541GA;5572CT;5730CT;5795GA;5856AG;5859TA;5914TC;5934GA;5967TC;5968GA;5994CT;6018TC;6048CT;6075TC;6210AG;6255AG;6303GA;6331AG | 145 |
| I | 86TA;114TC;129TC;174TC;231GA;237TC;360TC;396CT;483TC;519TC;534AT;544CT;550CT;693CT;744CT;774CT;777CT;786TC;801GA;822AC;851GA;879GA;881CT;922GA;957CT;966GA;997CT;1020TC;1044CT;1057CT;1067GA;1090TC;1098GA;1107CT;1119CT;1143CT;1179GA;1182AG;1227CT;1230AG;1238GA;1278TC;1299CT;1344CT;1350CT;1353CT;1407AT;1427GA;1470GA;1488GA;1500TC;1533CT;1563AG;1572CT;1602TA;1605AG;1626CT;1668AG;1710TC;1737TC;1764GA;1791AG;1812TC;1815GA;1821CT;1827TA;1830CT;1851AG;1929GA;2005GA;2019AG;2070AG;2142AG;2145CT;2176GA;2187CT;2199GA;2232CT;2322TA;2358CT;2406CT;2421TG;2424CT;2487GA;2520GA;2562AG;2569TC;2571GA;2628AG;2667GA;2724TC;2751CT;2826AG;2832TC;2835AG;2862CT;2910AG;2937CT;2943CT;2955TC;2958GA;2979AG;3036CT;3042GA;3072GA;3087AG;3090CT;3210CT;3237AG;3279AG;3283CT;3285GA;3304TC;3306AG;3318CT;3348GA;3426TC;3453TC;3465TC;3489TC;3591AG;3615TC;3642TA;3652CT;3699CT;3723AG;3768TC;3888GA;3894AG;3906CT;4014TC;4015GA;4108TC;4204TC;4206GA;4272TC;4323GA;4341GA;4392AC;4444TC;4527CT;4563TC;4584GA;4644CT;4659GA;4689AG;4704CT;4731CT;4794AG;4815AT;4827GA;4839TC;4905TC;4938TC;4953AG;4968AG;4977CT;5070CT;5079AG;5097AC;5157CT;5172AG;5187GA;5217TC;5253AT;5349AG;5406GA;5409GA;5433CT;5472TC;5490TC;5517GA;5526GA;5578CT;5596CT;5607TA;5664GA;5715AG;5730CT;5784GA;5800AC;5841AG;5916AG;5934GA;5967TC;5968GA;5970CT;5994CT;6024TA;6087TC;6093AG;6115GA;6126GA;6189TC;6231AG;6255AG;6258AG | 197 |
| J | 42GA;81GA;86TA;114TC;129TC;150TC;174TC;210AG;237TC;340TC;360TC;411AG;501TC;516TC;519TC;525GA;534AT;544CT;600TG;619AC;630CT;684AG;720CT;744CT;774CT;777CT;780TC;783CT;786TC;822AC;834AG;851GA;879GA;922GA;927GA;948AG;990TC;1053GA;1056AG;1057CT;1067GA;1090TC;1098GA;1119CT;1179GA;1182AG;1227CT;1230AG;1238GA;1299CT;1329TC;1344CT;1350CT;1353CT;1427GA;1455AG;1470GA;1488GA;1500TC;1524CT;1572CT;1584TA;1602TA;1605AG;1623CT;1626CT;1659TA;1661TA;1665GA;1668AG;1674GA;1704GA;1707TC;1710TC;1764GA;1812TC;1815GA;1821CT;1830CT;1839CT;2005GA;2019AG;2137TC;2160TA;2187CT;2193TC;2232CT;2262TC;2268AG;2358CT;2370GA;2406CT;2418AG;2421TG;2424CT;2487GA;2493CT;2520GA;2535TC;2544GA;2568GA;2571GA;2598TC;2604GA;2616CT;2619TC;2628AG;2637AG;2652TC;2724TC;2733GA;2751CT;2808GA;2835AG;2862CT;2886TC;2907TC;2910AG;2915AG;2943CT;3042GA;3072GA;3073CT;3090CT;3124TC;3168TC;3192CT;3210CT;3234TC;3237AG;3279AG;3283CT;3318CT;3348GA;3358CT;3390GA;3453TC;3465TC;3480GA;3489TC;3513GA;3642TA;3699CT;3723AG;3732AT;3768TC;3798AG;3888GA;3894AG;3895TC;3906CT;3936AG;3993TG;3996GA;4014TC;4015GA;4038CT;4092GA;4095GA;4101TC;4108TC;4272TC;4296AG;4323GA;4380AG;4389TC;4425GA;4428AG;4444TC;4482TC;4527CT;4557TA;4581CT;4584GA;4602GA;4623GC;4659GA;4689AG;4696TC;4710AG;4731CT;4746TC;4794AG;4815AT;4839TC;4905TC;4938TC;4953AG;4968AG;4977CT;5043GA;5070CT;5079AG;5133GA;5157CT;5172AG;5187GA;5217TC;5253AT;5285GA;5346GA;5349AG;5361CT;5406GA;5409GA;5418TC;5433CT;5490TC;5517GA;5596CT;5628TC;5637TC;5643GA;5664GA;5673CT;5715AG;5730CT;5784GA;5877CT;5934GA;5967TC;5968GA;5970CT;5980TA;6033TC;6060CT;6063CT;6075TC;6159AG;6231AG;6255AG;6279GA;6303GA;6327AG | 234 |
| K | 86TA;129TC;174TC;243CT;360TC;516TC;519TC;544CT;588CT;606GA;729TC;744CT;774CT;777CT;786TC;822AC;851GA;879GA;906AG;922GA;933CT;948AG;1041GA;1090TC;1098GA;1119CT;1179GA;1230AG;1234AG;1238GA;1344CT;1350CT;1353CT;1416GA;1427GA;1452TC;1470GA;1488GA;1500TC;1533CT;1569CT;1572CT;1573TC;1605AG;1626CT;1668AG;1671CT;1710TC;1716TC;1764GA;1812TC;1815GA;1821CT;1830CT;1932TC;2005GA;2019AG;2106TC;2109GA;2187CT;2232CT;2262TC;2358CT;2388AT;2406CT;2424CT;2508TC;2520GA;2554TC;2569TC;2571GA;2622GA;2628AG;2637AG;2658AG;2664GA;2838GA;2862CT;2880TA;2910AG;2937CT;2943CT;3042GA;3072GA;3090CT;3210CT;3283CT;3318CT;3369TA;3426TC;3453TC;3465TC;3489TC;3591AG;3621AT;3642TA;3684GA;3699CT;3738CT;3768TC;3894AG;3960TC;4023CT;4272TC;4332AG;4389TC;4419TC;4444TC;4455TC;4500AT;4584GA;4689AG;4731CT;4794AG;4815AT;4839TC;4938TC;4953AG;4968AG;4977CT;5070CT;5079AG;5091AG;5142AG;5187GA;5217TC;5253AT;5349AG;5406GA;5478TC;5490TC;5509CT;5517GA;5580TC;5596CT;5664GA;5730CT;5784GA;5787AG;5934GA;5967TC;5968GA;5970CT;6033TC;6144TC;6159AG;6231AG;6255AG;6303GA | 149 |
| L | 86TA;114TC;129TC;174TC;360TC;516TC;519TC;534AT;544CT;744CT;774CT;777CT;822AC;879GA;881CT;922GA;1020TC;1057CT;1090TC;1098GA;1107CT;1119CT;1179GA;1182AG;1227CT;1238GA;1344CT;1350CT;1353CT;1427GA;1470GA;1488GA;1500TC;1533CT;1572CT;1602TA;1605AG;1626CT;1668AG;1764GA;1812TC;1815GA;1821CT;2005GA;2106TC;2187CT;2232CT;2358CT;2406CT;2424CT;2487GA;2532GA;2571GA;2628AG;2637AG;2724TC;2751CT;2862CT;2910AG;2937CT;2943CT;3042GA;3072GA;3090CT;3237AG;3279AG;3283CT;3318CT;3348GA;3426TC;3453TC;3465TC;3489TC;3501CA;3642TA;3699CT;3723AG;3768TC;3894AG;3906CT;4108TC;4272TC;4444TC;4527CT;4584GA;4689AG;4731CT;4794AG;4815AT;4839TC;4905TC;4938TC;4953AG;4968AG;5070CT;5079AG;5172AG;5187GA;5217TC;5253AT;5349AG;5406GA;5409GA;5490TC;5517GA;5596CT;5730CT;5784GA;5934GA;5967TC;5970CT;5994CT;6159AG;6231AG;6255AG | 115 |
| M | 86TA;114TC;129TC;174TC;243CT;270CT;360TC;366TC;516TC;519TC;534AT;544CT;582TC;588CT;606GA;621GA;624CT;658CT;729TC;744CT;774CT;777CT;786TC;822AC;823AG;851GA;879GA;881CT;906AG;922GA;933CT;948AG;1020TC;1054GA;1057CT;1090TC;1098GA;1107CT;1119CT;1179GA;1182AG;1227CT;1230AG;1234AG;1238GA;1299CT;1329TC;1344CT;1350CT;1353CT;1427GA;1452TC;1467GA;1470GA;1488GA;1500TC;1533CT;1539AT;1569CT;1572CT;1573TC;1602TA;1605AG;1626CT;1668AG;1695TC;1710TC;1716TC;1764GA;1812TC;1815GA;1821CT;1830CT;1929GA;2005GA;2019AG;2079AG;2106TC;2109GA;2142AG;2187CT;2232CT;2262TC;2358CT;2388AT;2400GA;2406CT;2421TG;2424CT;2487GA;2508TC;2520GA;2554TC;2569TC;2571GA;2622GA;2628AG;2637AG;2658AG;2706CT;2724TC;2739GA;2751CT;2838GA;2858GA;2862CT;2910AG;2915AG;2937CT;2943CT;2944CA;2979AG;2994AG;3042GA;3072GA;3090CT;3138GA;3168TC;3210CT;3237AG;3279AG;3283CT;3318CT;3348GA;3369TA;3426TC;3432AG;3453TC;3465TC;3489TC;3537CT;3579GA;3585TC;3591AG;3621AT;3642TA;3699CT;3714TC;3723AG;3738CT;3768TC;3810AG;3894AG;3900AG;3903TG;3906CT;3924AG;3960TC;4014TC;4015GA;4023CT;4108TC;4272TC;4287CT;4323GA;4332AG;4362TC;4389TC;4419TC;4444TC;4455TC;4500AT;4527CT;4584GA;4689AG;4698GA;4722AG;4731CT;4737GA;4755TC;4794AG;4797CT;4815AT;4839TC;4905TC;4938TC;4944GA;4953AG;4968AG;4977CT;5025GC;5070CT;5079AG;5142AG;5157CT;5172AG;5187GA;5217TC;5253AT;5280CT;5349AG;5361CT;5406GA;5409GA;5414GA;5433CT;5478TC;5490TC;5509CT;5517GA;5580TC;5596CT;5628TC;5634CT;5664GA;5730CT;5784GA;5787AG;5923CT;5934GA;5964TA;5967TC;5968GA;5970CT;5994CT;6033TC;6054CT;6144TC;6159AG;6231AG;6255AG;6303GA;6312GA;6341AG | 224 |
| N | 60TC;86TA;114TC;129TC;174TC;216CT;327CT;330GT;360TC;516TC;519TC;522TC;534AT;543TC;544CT;549GA;570GA;738AT;744CT;765GA;774CT;777CT;786TC;822AC;879GA;906AG;912TC;922GA;933CT;948AG;969CT;972CT;1017TC;1020TC;1025TC;1057CT;1090TC;1098GA;1107CT;1119CT;1179GA;1182AG;1185AG;1224CT;1227CT;1230AG;1238GA;1257CT;1272AG;1290TC;1299CT;1338TC;1344CT;1350CT;1353CT;1427GA;1434CT;1458GA;1470GA;1473TC;1488GA;1500TC;1533CT;1572CT;1602TA;1605AG;1608AG;1626CT;1677CT;1710TC;1764GA;1812TC;1821CT;1830CT;1845CT;1866CT;1875CT;1932TA;1953TC;1990TC;2005GA;2070AG;2106TC;2115CT;2232CT;2262TC;2307TC;2334CT;2358CT;2406CT;2421TG;2424CT;2463TC;2475TC;2487GA;2493CT;2520GA;2568GA;2569TC;2571GA;2580TC;2589AG;2598TC;2628AG;2637AG;2724TC;2751CT;2862CT;2910AG;2937CT;2943CT;3042GA;3063AG;3072GA;3144GA;3210CT;3237AG;3279AG;3283CT;3318CT;3336CT;3354CT;3357CT;3390GA;3426TC;3453TC;3465TC;3489TC;3597TC;3621AG;3627CT;3630GA;3642TA;3699CT;3723AG;3753TC;3768TC;3786GA;3813CT;3814TC;3894AG;3906CT;4011AG;4108TC;4182TC;4207TC;4230TC;4257TC;4272TC;4323GA;4353GA;4362TC;4389TC;4444TC;4446AG;4509CT;4527CT;4584GT;4647AG;4671AG;4689AG;4704CT;4731CT;4794AG;4815AT;4902CT;4905TC;4938TC;4953AG;4968AG;4977CT;5013CT;5070CT;5172AG;5187GA;5253AT;5335AG;5391CT;5406GA;5409GA;5487TC;5490TC;5511GA;5517GA;5565CT;5580TC;5583GA;5596CT;5664GA;5679AG;5730CT;5754TC;5784GA;5793CT;5850AG;5883CT;5892CT;5904TC;5934GA;5967TC;5968GA;5970CT;5982GA;5994CT;6033TC;6075TC;6087TC;6099TC;6159AG;6252GA;6255AG;6315AT | 212 |
| O | 86TA;114TC;129TC;153CT;162CT;174TC;360TC;363AG;387CT;420AG;516TC;519TC;534AT;544CT;546GA;550CT;558CT;744CT;749GA;765GA;774CT;777CT;786TC;822AC;851GA;864TC;879GA;881CT;922GA;1020TC;1057CT;1090TC;1098GA;1107CT;1119CT;1143CT;1179GA;1182AG;1227CT;1230AG;1238GA;1295CG;1299CT;1344CT;1350CT;1353CT;1427GA;1470GA;1488GA;1500TC;1533CT;1542TC;1548AT;1572CT;1602TA;1605AG;1626CT;1647AG;1668AG;1710TC;1764GA;1806TC;1812TC;1815GA;1821CT;1830CT;1923GA;2005GA;2019AG;2070AG;2106TC;2142AG;2157AG;2187CT;2226CT;2232CT;2262TC;2358CT;2406CT;2421TG;2424CT;2454GA;2487GA;2499TC;2532GA;2554TC;2556GA;2569TC;2571GA;2628AG;2637AG;2646AG;2724TC;2751CT;2826AG;2835AG;2862CT;2886TC;2910AG;2937CT;2943CT;2979AG;3000GA;3042GA;3060TC;3072GA;3090CT;3237AG;3279AG;3283CT;3291TC;3304TC;3318CT;3348GA;3426TC;3453TC;3465TC;3489TC;3501CA;3642TA;3699CT;3723AG;3768TC;3867CT;3879TC;3894AG;3897GA;3906CT;3994AC;4014TC;4015GA;4053CT;4077AG;4108TC;4143TC;4173CT;4185CT;4272TC;4296AG;4444TC;4488TC;4527CT;4584GA;4689AG;4731CT;4794AG;4815AT;4839TC;4896AG;4905TC;4938TC;4953AG;4968AG;5014CT;5070CT;5079AG;5097AC;5157CT;5172AG;5187GA;5189AG;5217TC;5253AT;5328CT;5349AG;5406GA;5409GA;5472TC;5490TC;5517GA;5557TC;5562CT;5596CT;5664GA;5730CT;5784GA;5800AC;5934GA;5967TC;5968GA;5970CT;5994CT;6159AG;6231AG;6237TC;6249TC;6255AG;6258AG;6303GA;6326TC;6347GA | 191 |

**Table S4.** IgM antibody capture ELISA results showing Optical Density values with dilution buffer (OD1) and with Nucleoprotein added to the dilution buffer (OD2) measured at 450nm.

| **Tested Sample** | **OD1** | **OD2** | **Final_OD** | **Sample_Positivity** | **Interpretation** |
| --- | --- | --- | --- | --- | --- |
| DVS-333 | 0.057 | 0.783 | 0.726 | 87.05035971 | Positive |
| DVS-372 | 0.062 | 0.804 | 0.742 | 88.96882494 | Positive |
| DVS-356 | 0.079 | 1.23 | 1.151 | 138.0095923 | Positive |
| DVS-230 | 0.121 | 1.228 | 1.107 | 132.7338129 | Positive |
| DVS-321 | 0.067 | 1.277 | 1.21 | 145.0839329 | Positive |

**Table S5.** RVFV representative sequence data generators are cited in this file.

| Accession | Lineage | source | Strain | Country | Year | Authors |
| --- | --- | --- | --- | --- | --- | --- |
| NC_014396 | A | Human | ZH-548 | Egypt | 1977 | Bird,B.H., Khristova,M.L., Rollin,P.E., Ksiazek,T.G. and Nichol,S.T. |
| HM587040 | A | Human | MgH824 | Madagascar | 1979 | Grobbelaar,A.A., Weyer,J., Leman,P.A., Kemp,A., Paweska,J.T. and Swanepoel,R. |
| HM587045 | B | Ovine | S35 | Kenya | 1972 | Grobbelaar,A.A., Weyer,J., Leman,P.A., Kemp,A., Paweska,J.T. and Swanepoel,R. |
| DQ380197 | C | Human | Saudi 2000-10911 | Saudi Arabia | 2000 | Bird,B.H., Khristova,M.L., Rollin,P.E., Ksiazek,T.G. and Nichol,S.T. |
| DQ380196 | C | Human | Kenya 9800523 | Kenya | 1998 | Bird,B.H., Khristova,M.L., Rollin,P.E., Ksiazek,T.G. and Nichol,S.T. |
| JF311378 | C | Homo sapiens | 200803163 | Madagascar | Feb-2008 | Carroll,S.A., Reynes,J.M., Khristova,M.L., Andriamandimby,S.F., Rollin,P.E. and Nichol,S.T. |
| HQ009512 | C | Bovine | M48/08 | Madagascar | 2008 | Potgieter,C. |
| JQ820488 | C | Homo sapiens | Sudan 85-2010 | Sudan | Oct-2010 | Aradaib,I.E., Erickson,B.R., Elageb,R.M., Khristova,M.L., Carroll,S.A., Elkhidir,I.M., Karsany,M.E., Karrar,A.E., Elbashir,M.I. and Nichol,S.T. |
| DQ380221 | D | Human | 73HB1230 | Central African Republic | 1973 | Bird,B.H., Khristova,M.L., Rollin,P.E., Ksiazek,T.G. and Nichol,S.T. |
| DQ380212 | E | Homo sapiens | 74HB59 | Central African Republic | 1974 | Bird,B.H., Khristova,M.L., Rollin,P.E., Ksiazek,T.G. and Nichol,S.T. |
| DQ380211 | E | Human | 73HB1449 | Central African Republic | 1973 | Bird,B.H., Khristova,M.L., Rollin,P.E., Ksiazek,T.G. and Nichol,S.T. |
| HM587080 | E | Bovine | VRL1887/78 | Zimbabwe | 1978 | Grobbelaar,A.A., Weyer,J., Leman,P.A., Kemp,A., Paweska,J.T. and Swanepoel,R. |
| HM587080 | E | Bovine | VRL1887/78 | Zimbabwe | 1978 | Grobbelaar,A.A., Weyer,J., Leman,P.A., Kemp,A., Paweska,J.T. and Swanepoel,R. |
| HM587080 | E | Bovine | VRL1887/78 | Zimbabwe | 1978 | Grobbelaar,A.A., Weyer,J., Leman,P.A., Kemp,A., Paweska,J.T. and Swanepoel,R. |
| HM587080 | E | Bovine | VRL1887/78 | Zimbabwe | 1978 | Grobbelaar,A.A., Weyer,J., Leman,P.A., Kemp,A., Paweska,J.T. and Swanepoel,R. |
| HM587078 | E | Bovine | VRL1290A/78 | Zimbabwe | 1978 | Grobbelaar,A.A., Weyer,J., Leman,P.A., Kemp,A., Paweska,J.T. and Swanepoel,R. |
| HM587079 | E | Human | SPU44/85 | Zambia | 1985 | Grobbelaar,A.A., Weyer,J., Leman,P.A., Kemp,A., Paweska,J.T. and Swanepoel,R. |
| HM587079 | E | Human | SPU44/85 | Zambia | 1985 | Grobbelaar,A.A., Weyer,J., Leman,P.A., Kemp,A., Paweska,J.T. and Swanepoel,R. |
| HM587101 | F | Cx. zombaensis mosquito | Ar20364 | South Africa | 1981 | Grobbelaar,A.A., Weyer,J., Leman,P.A., Kemp,A., Paweska,J.T. and Swanepoel,R. |
| HM587101 | F | Cx. zombaensis mosquito | Ar20364 | South Africa | 1981 | Grobbelaar,A.A., Weyer,J., Leman,P.A., Kemp,A., Paweska,J.T. and Swanepoel,R. |
| HM587083 | G | Mansonia africana mosquito | ArB1976 | Central African Republic | 1969 | Grobbelaar,A.A., Weyer,J., Leman,P.A., Kemp,A., Paweska,J.T. and Swanepoel,R. |
| DQ380218 | G | Human | Hv-B375 | Central African Republic | 1985 | Bird,B.H., Khristova,M.L., Rollin,P.E., Ksiazek,T.G. and Nichol,S.T. |
| AF134502 | G | Micropteropus pusillus bat | An K6087 | Genetic reassortment of Rift Valley fever virus in nature |  | Sall,A.A., Zanotto,P.M., Sene,O.K., Zeller,H.G., Digoutte,J.P., Thiongane,Y. and Bouloy,M. |
| HM587084 | G | Hipposideros caffer bat | AnK3837 | Guinea | 1981 | Grobbelaar,A.A., Weyer,J., Leman,P.A., Kemp,A., Paweska,J.T. and Swanepoel,R. |
| AF134499 | G | Ae. vexans arabiensis mosquito | Ar D104769 | Genetic reassortment of Rift Valley fever virus in nature |  | Sall,A.A., Zanotto,P.M., Sene,O.K., Zeller,H.G., Digoutte,J.P., Thiongane,Y. and Bouloy,M. |
| HM587088 | H | Human | SA71/10 | South Africa | 2010 | Grobbelaar,A.A., Weyer,J., Leman,P.A., Kemp,A., Paweska,J.T. and Swanepoel,R. |
| HM587093 | H | Human | SA276/10 | South Africa | 2010 | Grobbelaar,A.A., Weyer,J., Leman,P.A., Kemp,A., Paweska,J.T. and Swanepoel,R. |
| HM587089 | H | Human | SA482/10 | South Africa | 2010 | Grobbelaar,A.A., Weyer,J., Leman,P.A., Kemp,A., Paweska,J.T. and Swanepoel,R. |
| HM587090 | H | Human | SA1221/10 | South Africa | 2010 | Grobbelaar,A.A., Weyer,J., Leman,P.A., Kemp,A., Paweska,J.T. and Swanepoel,R. |
| HM587097 | H | Human | SA373/10 | South Africa | 2010 | Grobbelaar,A.A., Weyer,J., Leman,P.A., Kemp,A., Paweska,J.T. and Swanepoel,R. |
| HM587108 | I | Ovine | An1830 | South Africa | 1956 | Grobbelaar,A.A., Weyer,J., Leman,P.A., Kemp,A., Paweska,J.T. and Swanepoel,R. |
| HM587109 | I | Ae. circumluteolus mosquito | Ar74 | South Africa | 1955 | Grobbelaar,A.A., Weyer,J., Leman,P.A., Kemp,A., Paweska,J.T. and Swanepoel,R. |
| DQ380222 | J | Bovine | 2269/74 | Zimbabwe | 1974 | Bird,B.H., Khristova,M.L., Rollin,P.E., Ksiazek,T.G. and Nichol,S.T. |
| DQ380194 | K | Homo sapiens | 2373/74 | Zimbabwe | 1974 | Bird,B.H., Khristova,M.L., Rollin,P.E., Ksiazek,T.G. and Nichol,S.T. |
| DQ380193 | K | RVFV strain Entebbe | Smithburn | Rift Valley fever virus genomics |  | Bird,B.H., Khristova,M.L. and Nichol,S.T. |
| DQ380191 | K | Eretmapodites spp. bat | Entebbe | Uganda | 1944 | Bird,B.H., Khristova,M.L., Rollin,P.E., Ksiazek,T.G. and Nichol,S.T. |
| HM587105 | K | Bovine | B314 | Kenya | 1962 | Grobbelaar,A.A., Weyer,J., Leman,P.A., Kemp,A., Paweska,J.T. and Swanepoel,R. |
| KX611606 | K | Homo sapiens | Beijing-01 | China | 22-Jul-2016 | Pan,Y., Cui,S., Sun,Y., Li,J., Lv,Y., Dou,X., Li,X., Tian,L., He,Z., Li,X., Chen,L. and Wang,Q. |
| HM587119 | M | Aedes spp. mosquito | Lunyo | Uganda | 1955 | Grobbelaar,A.A., Weyer,J., Leman,P.A., Kemp,A., Paweska,J.T. and Swanepoel,R. |
| HM587120 | M | Ae. circumluteolus mosquito | Ar118 | South Africa | 1955 | Grobbelaar,A.A., Weyer,J., Leman,P.A., Kemp,A., Paweska,J.T. and Swanepoel,R. |
| HM587110 | L | Human | H1739 | South Africa | 1975 | Grobbelaar,A.A., Weyer,J., Leman,P.A., Kemp,A., Paweska,J.T. and Swanepoel,R. |
| HM587118 | L | Bovine | KEN56/B2653/IB8 | Kenya | 1963 | Grobbelaar,A.A., Weyer,J., Leman,P.A., Kemp,A., Paweska,J.T. and Swanepoel,R. |
| JF784387 | L | Bovine | 35/74 | South Africa | 1974 | Kortekaas,J., Oreshkova,N., Cobos-Jimenez,V., Vloet,R.P., Potgieter,C.A. and Moormann,R.J. |
| DQ380189 | L | Human | SA-75 | South Africa | 1975 | Bird,B.H., Khristova,M.L., Rollin,P.E., Ksiazek,T.G. and Nichol,S.T. |
| HM587111 | L | Bovine | VRL763/70 | Zimbabwe | 1970 | Grobbelaar,A.A., Weyer,J., Leman,P.A., Kemp,A., Paweska,J.T. and Swanepoel,R. |
| DQ380186 | N | Human | OS-1 | Mauritania | 1987 | Bird,B.H., Khristova,M.L., Rollin,P.E., Ksiazek,T.G. and Nichol,S.T. |
| DQ380183 | N | Human | OS-9 | Mauritania | 1987 | Bird,B.H., Khristova,M.L., Rollin,P.E., Ksiazek,T.G. and Nichol,S.T. |
| DQ380185 | N | Human | OS-8 | Mauritania | 1987 | Bird,B.H., Khristova,M.L., Rollin,P.E., Ksiazek,T.G. and Nichol,S.T. |
| DQ380184 | N | Human | OS-3 | Mauritania | 1987 | Bird,B.H., Khristova,M.L., Rollin,P.E., Ksiazek,T.G. and Nichol,S.T. |
| DQ380187 | N | Ae. cuminsi mosquito | ARD-38388 | Burkina Faso | 1983 | Bird,B.H., Khristova,M.L., Rollin,P.E., Ksiazek,T.G. and Nichol,S.T. |
| HM587125 | O | Ovine | SA51 | South Africa | 1951 | Grobbelaar,A.A., Weyer,J., Leman,P.A., Kemp,A., Paweska,J.T. and Swanepoel,R. |
| DQ380195 | O | Ovine | SA-51 (Van Wyck) | South Africa | 1951 | Bird,B.H., Khristova,M.L., Rollin,P.E., Ksiazek,T.G. and Nichol,S.T. |
| DQ380209 | A | Bovine | 2250/74 | Zimbabwe | 1974 | Bird,B.H., Khristova,M.L., Rollin,P.E., Ksiazek,T.G. and Nichol,S.T. |
| DQ380199 | A | Human | T-46 (228113) | Egypt | 1977 | Bird,B.H., Khristova,M.L. and Nichol,S.T. |
| DQ380208 | A | RVF isolate ZH548 | MP-12 | Rift Valley fever virus genomics |  | Bird,B.H., Khristova,M.L. and Nichol,S.T. |
| DQ380205 | A | Ovine | ZS-6365 | Egypt | 1979 | Bird,B.H., Khristova,M.L., Rollin,P.E., Ksiazek,T.G. and Nichol,S.T. |

**Supplementary Figures**


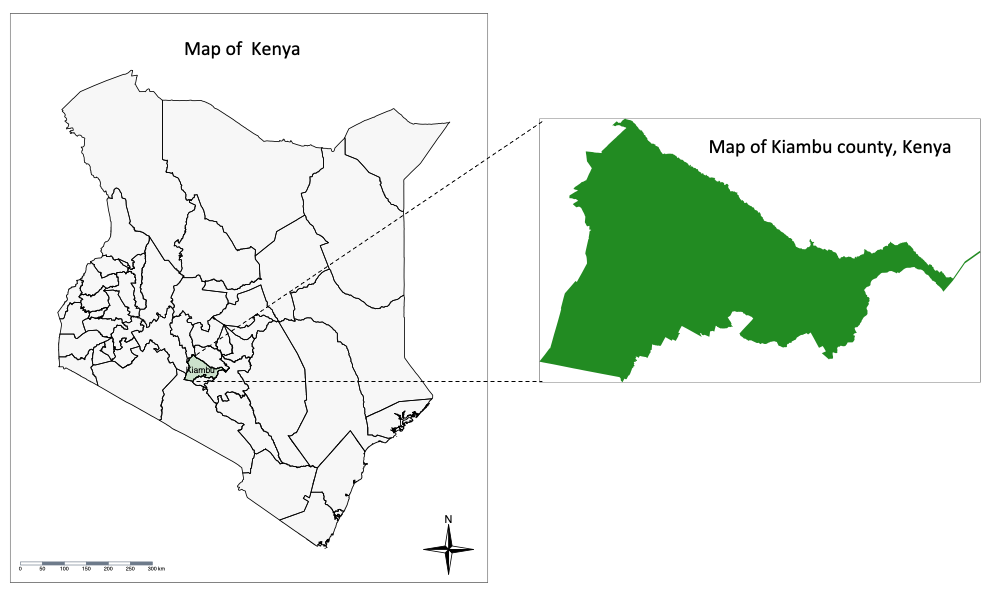


**Figure S1.** Sampling areas in Kenya where suspected cases of Rift Valley fever were reported.


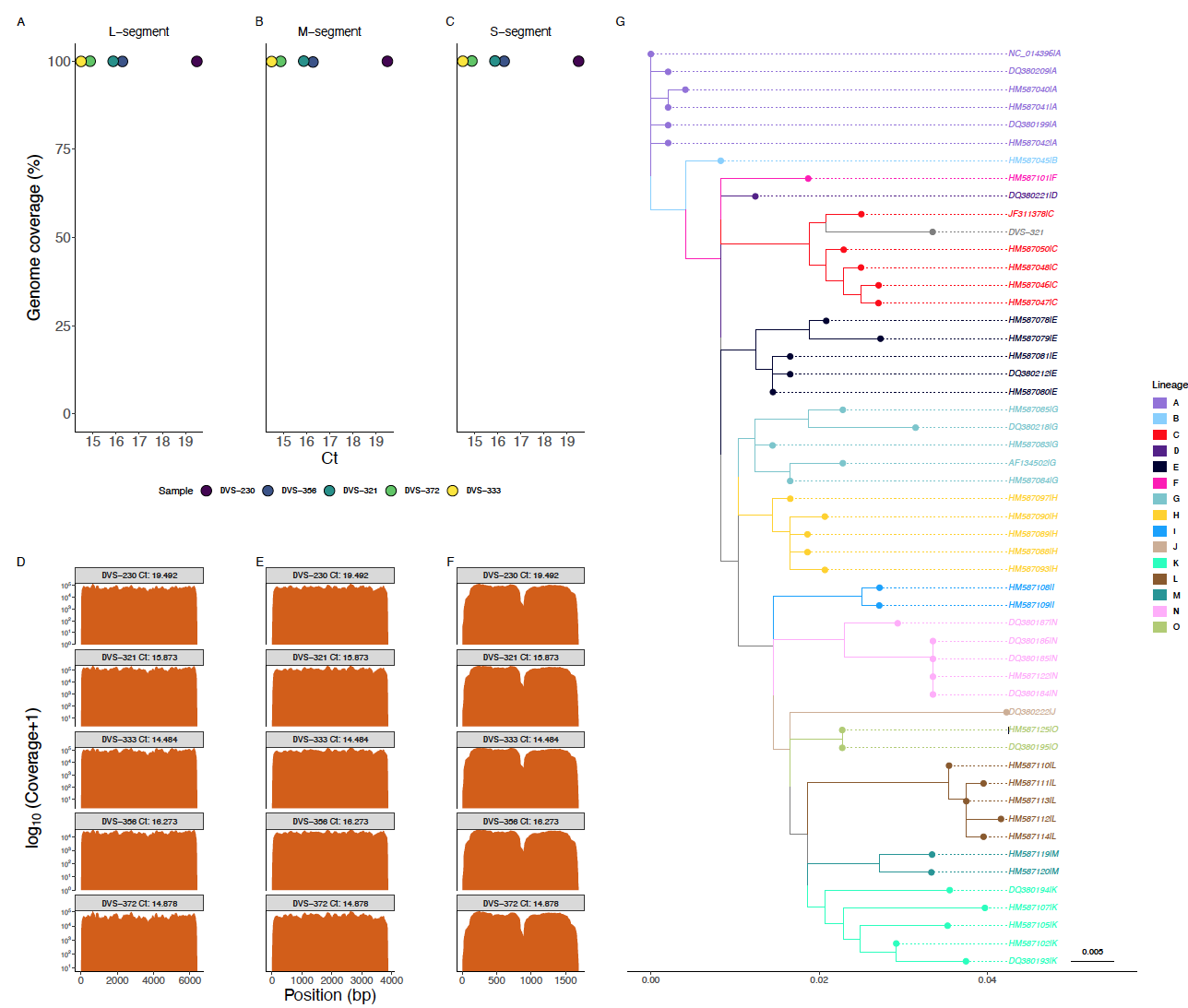


**Figure S2. Genome sequencing and phylogenetic analysis. (A-C)** RT-qPCR cycle threshold (Ct) values (*x*-axis) plotted against percent genome covered (*y*-axis) for L, M and S-segments of the RVFV genome. **(D-F), Genome** coverage (log10 transformed) along genomic positions in base pairs. **(G)** Maximum likelihood phylogenetic tree indicating the different clades corresponding to the fifteen major lineages and showing where a query sequence (DVS-321) is clustered in the tree.

References:

1. Bird BH, Bawiec DA, Ksiazek TG, Shoemaker TR, Nichol ST. Highly Sensitive and Broadly Reactive Quantitative Reverse Transcription-PCR Assay for High-Throughput Detection of Rift Valley Fever Virus. J Clin Microbiol. 2007;45:3506–13.

2. Andrews S. FastQC: a quality control tool for high throughput sequence data. 2010. http://www.bioinformatics.babraham.ac.uk/projects/fastqc.

3. Chen S, Zhou Y, Chen Y, Gu J. fastp: an ultra-fast all-in-one FASTQ preprocessor. Bioinformatics. 2018;34:i884–90.

4. Li H, Durbin R. Fast and accurate short read alignment with Burrows–Wheeler transform. Bioinformatics. 2009;25:1754–60.

5. Li H, Handsaker B, Wysoker A, Fennell T, Ruan J, Homer N, et al. The Sequence Alignment/Map format and SAMtools. Bioinformatics. 2009;25:2078–9.

6. Quinlan AR, Hall IM. BEDTools: a flexible suite of utilities for comparing genomic features. Bioinformatics. 2010;26:841–2.
